# Host and bacterial urine proteomics might predict treatment outcomes for immunotherapy in advanced non-small cell lung cancer patients

**DOI:** 10.3389/fimmu.2025.1543817

**Published:** 2025-04-14

**Authors:** David Dora, Peter Revisnyei, Alija Pasic, Gabriella Galffy, Edit Dulka, Anna Mihucz, Brigitta Roskó, Sara Szincsak, Anton Iliuk, Glen J. Weiss, Zoltan Lohinai

**Affiliations:** ^1^ Department of Anatomy, Histology and Embryology, Semmelweis University, Budapest, Hungary; ^2^ Department of Telecommunications and Media Informatics, Budapest University of Technology and Economics, Budapest, Hungary; ^3^ HUN-REN-BME Information Systems Research Group, Budapest, Hungary; ^4^ County Hospital of Torokbalint, Torokbalint, Hungary; ^5^ Translational Medicine Institute, Semmelweis University, Budapest, Hungary; ^6^ Tymora Analytical Operations, West Lafayette, IN, United States; ^7^ Department of Medicine, UMass Chan Medical School, Worcester, MA, United States

**Keywords:** NSCLC, immunotherapy, gut microbiome, EV protein, urine proteome, machine learning

## Abstract

**Introduction:**

Urine samples are non-invasive approaches to study potential circulating biomarkers from the host organism. Specific proteins cross the bloodstream through the intestinal barrier and may also derive from gut microbiota. In this study, we aimed to evaluate the predictive role of the host and bacterial urine extracellular vesicle (EV) proteomes in patients with non-small cell lung cancer (NSCLC) treated with anti-PD1 immunotherapy.

**Methods:**

We analyzed the urine EV proteome of 33 advanced-stage NSCLC patients treated with anti-PD1 immunotherapy with LC-MS/MS, stratifying patients according to long (>6 months) and short (≤6 months) progression-free survival (PFS). Gut microbial communities on a subcohort of 23 patients were also analyzed with shotgun metagenomics. Internal validation was performed using the Random Forest (RF) machine learning (ML) algorithm. RF was validated with a non-linear Bayesian ML model. Gene enrichment, and pathway analysis of host urine proteins were analyzed using the Reactome and Gene Ontology databases.

**Results:**

We identified human (n=3513), bacterial (n=2647), fungal (n=19), and viral (n=4) proteins. 186 human proteins showed differential abundance (p<0.05) according to PFS groups, 101 being significantly more abundant in patients with short PFS and n=85 in patients with long PFS. We found several pathways that were significantly enriched in patients with short PFS (vs long PFS). Multivariate Cox regression showed that human urine proteins MPP5, IGKV6-21, NT5E, and KRT27 were strongly associated with long PFS, and LMAN2, NUTF2, NID1, TNC, IGF1, BCR, GPHN, and PPBP showed the strongest association with short PFS. We revealed that an increased bacterial/host protein ratio in the urine is more frequent in patients with long PFS. Increased abundance of *E. coli* and *E. faecalis* proteins in the urine positively correlates with their gut metagenomic abundance. RF ML model supported the reliability in predicting PFS for critical human urine proteins (AUC=0.89), accuracy (95%) and Bacterial proteins (AUC=0.74).

**Conclusion:**

To our knowledge, this is the first study to depict the predictive role of the host and bacterial urine proteome in anti-PD1-treated advanced NSCLC.

## Introduction

Anti-programmed death ligand-1 (PD-L1) immunotherapy with and without chemotherapy are now the standard of care in multiple cancers, including front-line therapy in advanced-stage non-small cell lung cancer (NSCLC) (1). The five-year overall survival (OS) increased to 20% in unselected patients and up to 40% in PD-L1^high^-expressing patients (1–6). Others showed that single agent anti-PD immunotherapy can be extended as first-line therapy to patients with advanced stage tumors and low-PD-L1 TPS (7). Clinical evidence shows that more than 50% of PD-L1^high^-expressing patients still do not respond to PD-1/PD-L1 blockade (8). Thus, there is a need to develop novel biomarkers to enhance efficacy.

Urinary tests have been used as cost-effective and noninvasive tools for the screening, diagnosis, and monitoring of various conditions. In addition to the assessment of bladder and other genito-urinary cancers (9, 10), urine can also indicate remote malignancies not directly associated with the urinary tract, similar to liquid biopsies, or analyzing of cell-free circulating DNA passing through glomerular filtration from the bloodstream (9). Mass spectroscopy can reveal a plethora of proteomic biomarkers examined in centrifuged urine for pancreatic, (11), lung, (12), colorectal, and gastric cancer (13). Quantitative analysis of urinary metabolites provided predictive models for the diagnosis of clear cell renal carcinoma (14), and to distinguish cholangiocarcinoma from periductal fibrosis (15).

Circulating acellular components of innate immunity, such as proteins of acute phase and complement activation can yield valuable insights about the course of the disease and the response to treatment, modulated by direct effects on tumor cells and by supporting a cancer-abetting microenvironment (16). Recent studies reported that mass-spectrometry-based plasma proteomic signatures could predict survival in immune checkpoint inhibitor (ICI)-treated advanced-stage NSCLC patients (17–19). However, to date, we are unaware of a published study on urine biomarkers to predict ICI efficacy in NSCLC. In addition, recent discoveries indicate that extracellular vesicles (EVs) provide an effective and ubiquitous method for intercellular communication, stimulation of immune system, removal of harmful materials, etc. (20–22). As these are shed into every biological fluid and embody a good representation of their parent cell, analysis of the EV cargo has great promise for biomarker discovery and disease diagnosis (23). Studies analyzing plasma EV signatures, including PD-L1 mRNA (24), specific microRNAs (25, 26), long mRNAs (27) and proteins (28) successfully established robust EV signatures associated with ICI-response.

Understanding of an intriguing association between the gut microbiome and ICI outcomes (efficacy and toxicity) has been unfolding in recent years (29, 30). Both in metastatic melanoma and NSCLC, multiple studies reported the linkage between microbial metagenomic and metatatranscriptomic signatures and ICI efficacy (31–39). The introduction of tumor and plasma microbial DNA analysis as a diagnostic approach and predictive factor in cancer was already proposed (40, 41). However, bacterial proteomic signatures in the urine have not been comprehensively studied relative to lung cancer.

In this study, we analyzed the host and microbial proteomes from urinary EVs of 33 advanced-stage NSCLC patients treated with anti-PD1 immunotherapy and established a comprehensive human and bacterial proteome profile for patients with long (>6 months) and short (≤6 months) PFS, and used machine learning (ML) algorithm Random Forest (RF) to internally validate our findings.

## Materials and methods

### Study population and treatments

A total of n=33 advanced-stage NSCLC patients treated with ICI were enrolled in this study who received standard-of-care second line nivolumab (anti-PD1) monotherapy (n=16), or first line pembrolizumab (anti-PD1) monotherapy (n=10) and atezolizumab (anti-PD-L1) monotherapy (n=1) or durvalumab-based (anti-PD-L1) chemotherapy-immunotherapy (CHT+IO) combination (n=6) (**Supplementary Table 2**). Immunotherapeutic agents were administered first line, if PD-L1 Tumor Proportion Score (TPS) was ≥50% and second line if PD-L1 TPS was <50%. All treatments were administered between 2019 and 2020 at the County Hospital of Pulmonology, Torokbalint, Hungary. For patients receiving chemo-immunotherapy (CHT+IO), the regimen included pemetrexed + carboplatin with durvalumab or pembrolizumab. Patients treated subsequent line with ICI received standard first-line platinum-based doublet therapy, selected per clinical guidelines and physician discretion. All patients included in our cohort were diagnosed with advanced-stage NSCLC (Stage IIIB/IV) with histologically confirmed adenocarcinoma (ADC), squamous cell carcinoma, and non-small cell lung carcinoma not otherwise specified (NSCLC-NOS). The clinical TNM (Tumor, Node, Metastasis) stage was determined according to the Union for International Cancer Control (8th edition). Baseline urine samples were collected before or within one week after the first cycle of immunotherapy. Follow-up urine samples were collected from n=17 patients 120 (± 7 days) after the first cycle. All patients underwent routine clinical urine testing for pyuria, hematuria, and bacteriuria and were screened for symptoms of urinary tract infections (fever, discharge, dysuria). Patients with suspected UTIs were excluded from the study.

Baseline stool samples were obtained from n=23 patients during baseline urine sample collection and forwarded for microbiome genome analysis (shotgun metagenomics). Clinicopathological data were collected at diagnosis, including age, gender, stage, histology, BMI, the diagnosis of COPD, chemotherapy administration [first line platinum based doublet without first line ICI (chemo-treated) vs first line single agent ICI (chemo-naïve)], tumor PD-L1 expression (PD-L1 TPS <50% vs ≥50%) and progression-free survival (PFS). PFS was calculated from the time of the first immunotherapy cycle until progression. Since there were only two non-smoker patients in our cohort (5.23%), we could not analyze smoking status. The date of the last follow-up included in this analysis was June 2022. All treatments were conducted under the contemporary National Comprehensive Cancer Network guidelines. Platinum based doublet chemotherapy was administered to patients in first line followed by a single agent ICI. The first line single agent ICI drug administration was covered by the insurance in the study period if PD-L1 TPS was above 50%.

Patients were classified based on short-term (≤6 months) versus long-term PFS (>6 months). **Table 1** shows the clinicopathological characteristics of our study cohort. **Supplementary Table 1** shows the inclusion and exclusion criteria of patients. **Supplementary Table 2** shows type of immunotherapies administered to patients.

**Table 1:** Clinicopathological characteristics of the patient cohort.

	Long PFS N=22 (67%)	Short PFS N=11 (33%)	p-value
Age [years (mean)]	60.23	58.8	0.173
Gender			
male [48% (n=16)] female [52% (n=17)]	41% (n=9) 59% (n=13)	64% (n=7) 36% (n=4)	0.281
Histology			
ADC [73% (n=24)] SCC [27% (n=9)]	77% (n=17) 23% (n=5)	64% (n=7) 36% (n=4)	
Stage			
Stage IIIb [21% (n=7)] Stage IV [79% (n=33)]	12% (n=4) 88% (n=18)	27% (n=3) 73% (n=7)	
ICI Response (R) at 3 months			
Response^1^ [82% (n=27)] Non-response^2^ [18% (n=6)]	100% (n=22) 0% (n=0)	45% (n=5) 55% (n=6)	<0.001***
PFS [months, (median)]	12.07	3.47	<0.001***
Chemotherapy			
CHT-treated [54% (n=18)] CHT-naive [46% (n=15)]	50% (n=11) 50% (n=11)	64% (n=7) 36% (n=4)	0.712
PD-L1 TPS >50% [36% (n=12)] ≤50% [64% (n=21)]	36% (8) 64% (14)	36% (4) 64% (7)	>0.999
Smoking [pack-years (mean)	37.12	40.4	0.096
COPD comorbidity present [36% (n=12)] not present [36% (n=21)]	41% (9) 59% (13)	27% (3) 73% (8)	0.702
BMI >30 kg/m^2^ [36% (n=12)] ≤30 kg/m^2^ [36% (n=12)]	36% (8) 64% (14)	36% (4) 64% (7)	>0.999

*p < 0.05, **p < 0.01, ***p < 0.001.

Response^1^: complete response (CR), partial response (PR), stable disease (SD).

Non-response^2^: progressive disease (PD).

### PD-L1 immunohistochemistry

Tumor samples retrieved by lung biopsy were available for PD-L1 immunohistochemistry (IHC) for all 33 advanced-stage NSCLC patients. For IHC staining, 4-µm-sections were cut from formalin-fixed-paraffin-embedded (FFPE) blocks. Staining was carried out on a Leica Bond RX autostainer using rabbit monoclonal antibody for PD-L1 diluted 1:300 (CST, cat: 13684S). Slides were stained with the Bond Polymer Refine Detection kit (#DS9800) and Leica IHC Protocol F, and epitope retrieval was carried out for twenty minutes at low pH. Slides were cleared and dehydrated on a Tissue-Tek Prisma platform before being coverslipped using a Tissue-Tek Film coverslipper. An experienced and certified histopathologist evaluated PD-L1 expression according to the FDA-approved TPS scoring system. Patients were classified as PD-L1-high (TPS ≥ 50%) or low (TPS < 50% percentile) expression.

### Preparation of EV samples

As previously described, EVs from 900 µL of each urine sample were captured and processed by Tymora Analytical Operations (West Lafayette, IN) using magnetic EVtrap beads (42). EV samples were characterized according to MISEV2023 recommendations.

Urinary EVs were isolated and analyzed using the EVtrap (Extracellular Vesicles Total Recovery and Purification) method, a high-efficiency magnetic bead-based affinity approach developed by Tymora Analytical Operations. This method captures EVs through their lipid bilayer interactions with amphiphilic beads, ensuring high recovery and minimal contamination from soluble urinary proteins. EVtrap has been extensively validated in prior studies, demonstrating >95% recovery efficiency, with seven times greater capture of CD9-positive EVs compared to ultracentrifugation (43, 44). The method has also been shown to significantly reduce the presence of common urinary contaminants, such as albumin and Tamm-Horsfall protein, through optimized bead-based binding and elution conditions. EV-specific protein markers CD9, CD63, and CD81 were verified using Western blot.

### Extraction of EV proteins

The isolated and dried EV samples were lysed to extract proteins using the phase-transfer surfactant (PTS) aided procedure (42). The proteins were reduced and alkylated by incubation in 10 mM tris(2-carboxyethyl)phosphine (TCEP) and 40 mM chloroacetamide (CAA) for 10 min at 95°C. The samples were diluted fivefold with 50 mM triethylammonium bicarbonate and digested with Lys-C (Wako) at 1:100 (wt/wt) enzyme-to-protein ratio for 3 h at 37°C. Trypsin was added to a final 1:50 (wt/wt) enzyme-to-protein ratio for overnight digestion at 37°C. Next, we removed the PTS surfactants from the samples, the samples were acidified with trifluoroacetic acid (TFA) to a final concentration of 1% TFA, and ethyl acetate solution was added at a 1:1 ratio. The mixture was vortexed for 2 min and then centrifuged at 16,000 × g for 2 min to obtain aqueous and organic phases. The organic phase (top layer) was removed, and the aqueous phase was collected. This step was repeated once more. According to the manufacturer's instructions, the samples were dried in a vacuum centrifuge and desalted using Top-Tip C18 tips (Glygen). A portion of each sample was used to determine peptide concentration with Pierce Quantitative Colorimetric Peptide Assay. The samples were dried completely in a vacuum centrifuge and stored at -80°C.

### LC-MS/MS analysis

Each dried peptide sample was dissolved at 0.1 μg/μL in 0.05% trifluoroacetic acid with 3% (vol/vol) acetonitrile. Ten μL of each sample was injected into an Ultimate 3000 nano UHPLC system (Thermo Fisher Scientific). Peptides were captured on a 2-cm Acclaim PepMap trap column and separated on a heated 50-cm column packed with ReproSil Saphir 1.8 μm C18 beads (Dr. Maisch GmbH). The mobile phase buffer consisted of 0.1% formic acid in ultrapure water (buffer A) with an eluting buffer of 0.1% formic acid in 80% (vol/vol) acetonitrile (buffer B) run with a linear 60-min gradient of 6–30% buffer B at a flow rate of 300 nL/min. The UHPLC was coupled online with a Q-Exactive HF-X mass spectrometer (Thermo Fisher Scientific). The mass spectrometer was operated in the data-dependent mode, in which a full-scan MS (from m/z 375 to 1,500 with a resolution of 60,000) was followed by MS/MS of the 15 most intense ions (30,000 resolution; normalized collision energy - 28%; automatic gain control target (AGC) - 2E4, maximum injection time - 200 ms; 60sec exclusion].

### LC-MS data processing

The raw files were searched directly against the human, bacterial, fungal and viral Uniprot databases with no redundant entries, using Byonic (Protein Metrics) and Sequest search engines loaded into Proteome Discoverer 2.3 software (Thermo Fisher Scientific). MS1 precursor mass tolerance was set at 10 ppm, and MS2 tolerance was set at 20 ppm. Search criteria included a static carbamidomethylation of cysteines (+57.0214 Da) and variable modifications of oxidation (+15.9949 Da) on methionine residues and acetylation (+42.011 Da) at the N terminus of proteins. The search was performed with full trypsin/P digestion, allowing a maximum of two missed cleavages on the peptides analyzed from the sequence database. The false-discovery rates of proteins and peptides were set at 0.01. All protein and peptide identifications were grouped, and any redundant entries were removed. Unique peptides and unique master proteins were reported.

### Label-free quantitation analysis

All data were quantified using the label-free quantitation node of Precursor Ions Quantifier through the Proteome Discoverer v2.3 (Thermo Fisher Scientific). For the quantification of proteomic data, the intensities of peptides were extracted with initial precursor mass tolerance set at ten ppm, minimum number of isotope peaks as 2, maximum ΔRT of isotope pattern multiplets – 0.2 min, PSM confidence FDR of 0.01, with hypothesis test of ANOVA, maximum RT shift of 5 min, pairwise ratio-based ratio calculation, and 100 as the maximum allowed fold change. The abundance levels of all peptides and proteins were normalized using the total peptide amount normalization node in the Proteome Discoverer. For calculations of fold-change between the groups of proteins, total protein abundance values were added together, and the ratios of these sums were used to compare proteins within different samples.

### Metagenomic sequencing

Within seven days of obtaining signed informed consent from the patients, baseline stool samples were collected before or after the first ICI infusion. On the day of collection, the samples were frozen at -80°C until they were separated and sequenced. We utilized 100 mg stool sample in ZR Bashing Bead Lysis Tubes with ZymoBIOMICS 96 MagBead DNA kit for entire DNA extraction, followed by 40 minutes of continuous bead beating and 1 minute of centrifugation at 10,000 x g. 200:l supernatant was shaken for 10 minutes with 25:l ZymoBIOMICSTM MagBinding Beads. After removing the supernatant from the tubes and placing them on a magnetic rack, 500:l ZymoBIOMICSTM MagBinding Buffer was added to each sample and stirred for 1 minute. The beads were pelleted and washed twice for 1 minute each with 500:l of ZymoBIOMICSTM MagWash 1 and 900:l of ZymoBIOMICSTM MagWash 2. The beads were dried for 10 minutes at 55°C before being eluted in 50 l RNAse/DNAse-free water. The DNA concentration was determined using a Qubit fluorimeter.

According to the manufacturer's recommendations, 65 ng of each sample was utilized as input for library preparation by the KAPA HyperPlus kit, with size selection for 200bp peak fragment size (TapeStation 2200, High Sensitivity D1000 ScreenTape®). The samples were sequenced on the NextSeq500 platform using 2x150bp read pairs and 10M read pairs.

### Microbial taxonomic profiling

The readings were adaptor-trimmed and quality-filtered to achieve a mean Q-score of 30 or above. FastQC was used to run a quality check, and it passed each sequence quality score per base N content and per adapter content. ( *http://www.bioinformatics.babraham.ac.uk/projects/fastqc* ). Kraken2 (version 2.0.8) (45) and the MiniKraken2 database were utilized for taxonomic assignment. The output files were combined into a data matrix using the combine kreports.py tool from KrakenTools (v1.2). The read counts were normalized using the smallest sample as the minimum depth and inclusion criteria of at least one read in at least one sample per taxon. A considerable proportion of the readings had been rendered as unclassified (mean=0.58, SD=0.086). For statistical analysis, the findings were stratified by taxa. Taxa that did not contribute at least 0.01 percent of overall abundance in the entire cohort were eliminated from the study prior to rarefaction. In subsequent analyses, only taxa from the domains *Bacteria* and *Archaea* were included; all viral and eukaryotic taxonomic units were omitted. The centered log-ratio (CLR) transformation method was used to further normalize rarefied abundance implemented in sci-kit-bio (46). CLR-transformation transforms sample vectors based on the logarithm of the ratio between the individual elements and the geometric mean of the vector.

### Machine learning models

Using the scikit-learn (1.1.2) python (3.10.6) package, multiple Random Forest (RF) models with stratified five-fold cross-validation were developed for binary classification. Binary classifications of PFS (long vs. short), PD-L1 (high vs low), and chemotherapy (naive vs. treated) were employed as independent targets. Individual models were trained using datasets from specific human and bacterial metabolites. Both datasets were used singly and in combination. The optimal RF model was identified based on its hyperparameters. The evaluation of these hyperparameters was conducted at grid points representing combinations of hyperparameters, chosen within specific intervals. At each gridpoint, the median of the mean AUC (Area Under the Curve) scores from cross-validation was calculated, and the model exhibiting the highest median value was selected as the final model. The primary hyperparameters targeted for optimization were the forest's number of trees, trees' maximum depth, and the minimum number of samples necessary for splitting an internal node. The best set of hyperparameters was determined based on the AUC score and ROC (Receiver Operating Characteristic) curve, which were assessed after the stratified 5-fold cross-validation.

Bayesian Additive Regression Trees (BART) was implemented using the dbarts package in R to classify patients into Long vs. Short PFS based on host urine EV protein abundances. The model was trained using a prior distribution to control complexity and avoid overfitting. Posterior probability estimates were generated, and model performance was assessed via AUC-ROC, posterior inclusion probabilities, and partial dependence plots. Leave-one-out cross-validation (LOO-CV) was performed using the loo package to estimate predictive accuracy. Feature importance was determined based on posterior inclusion probabilities, and non-linear effects were visualized with partial dependence plots (PDPs). All analyses were conducted in R 4.x with ggplot2 for visualization.

### Data preprocessing, pathway- and statistical analyses

We used the Shapiro-Wilk test to decide which statistical tools were applicable to the dataset. Accordingly, non-parametric tests were used in our research. In order to examine the relationships between the protein abundances and the PFS values, multiple Spearman's correlation tests were executed, and results were displayed on Volcano plots. To explore differentially abundant proteins in distinct patient groups (short vs. long PFS, CHT-naive vs. CHT-treated, and PD-L1 high vs. PD-L1 low), Wilcoxon rank-sum (WRS) tests were performed. Proteins with the 10 most significant p-values in every patient group were displayed in bar charts. We excluded the proteins with fewer measurement data points than a given threshold (20).

Pathway analyses were performed separately from the human and bacterial protein pool narrowed down to include proteins only with significant correlation with PFS (in months) (r(s) > [0.3], p < 0.05), or significant WRS test between patients with short vs long PFS. Only proteins which had more than 20 data points were included. For human pathway analyses, the Reactome and Gene Ontology (GO) databases were utilized, and data was generated with the WebGestalt software package. Over-representation (ORA) analysis was used to determine enrichment ratios and false discovery rate (FDR) for every pathway. For bacterial pathway analyses, the UniProt ID mapping tool was used to translate ascension numbers to bacterial gene IDs. Next, the FUNAGE-Pro functional analysis pipeline was used (47) to perform gene enrichment analyses based on the Kyoto Encyclopedia of Genes and Genomes (KEGG). Benjamini–Hochberg multiple testing correction was applied for all pathway analyses to calculate the final p-values.

For multivariate Cox-proportional hazard regression, the analysis was two-sided, with a significance threshold of=0.05. The predictive value of all urine proteins was tested with confounders' gender, chemotherapy, PD-L1 IHC expression, the presence of COPD, and BMI (see **Table 1**). Harrel's C-index was calculated to assess the quality of fit of our multivariate model that performed above 0.7 (fair) in all analyses.

Principal Component Analysis (PCA) was performed on human and bacterial proteins pooled, which had more than 20 data points, correlated significantly with PFS (r(s) > [0.3], p < 0.05), or exhibited a significant WRS test between patients with short vs long PFS. Then, the first two principal components were utilized for clustering the patients with the K-means clustering algorithm. The created clusters are displayed on scatter plots, and we visualized the clusters by multiple clinical properties of the patients.

## Results

**Figure 1:**
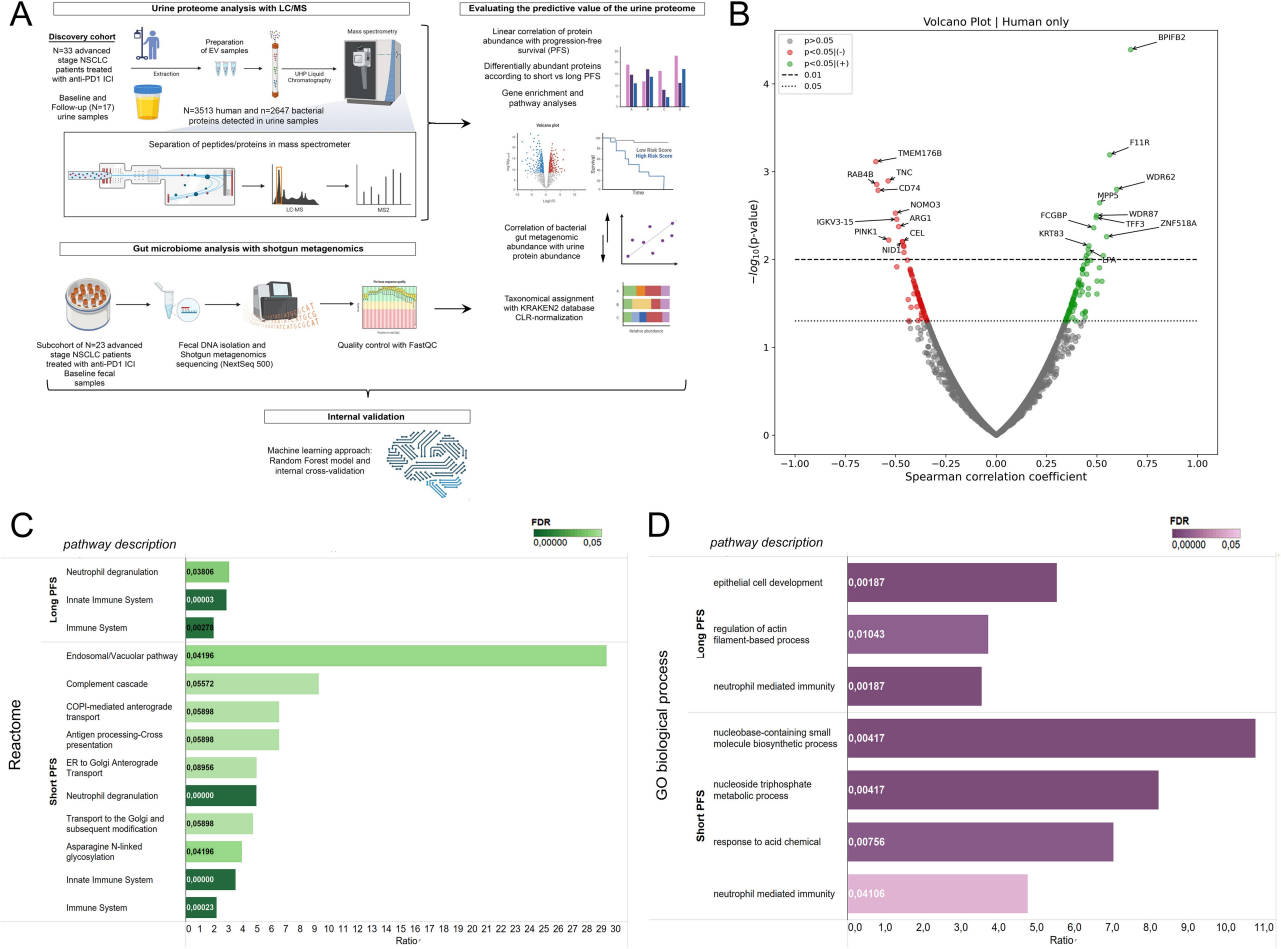
Correlation of the human urine EV proteome with progression-free survival and biological pathways. **(A)** Illustrated flowchart shows the study design, cohorts, and experimental procedures. **(B)** The volcano plot displays urine EV proteins according to their correlation with PFS in months. Spearman's correlation coefficient is shown in the X axis and the corresponding -*log*10 (p-value) in the Y axis. Non-significant proteins are grey; those showing significant positive correlation (p<0.05) with PFS are green, and those showing significant negative correlation are red. The dotted line indicates p<0.05-, the dashed line indicates p<0.01 threshold. **(C, D)** Pathway analyses using the Reactome and GO biological process databases were performed with ORA from whole exome datasets. Only proteins with significant correlation with PFS (in months) (r(s) > [0.3], p < 0.05), OR significant WRS test between patients with short vs long PFS were included. For multiple testing, Benjamini-Hochberg adjustment was used; False Discovery Rate (FDR) is indicated with color tone and labels in the horizontal bar charts. The enrichment ratio is displayed on the X-axis. Affinity Propagation was used to eliminate redundant pathways, and FDR-values were considered significant with p<0.1.

Our patient cohort included 33 advanced-stage lung cancer patients treated with anti-PD1 ICI. There were 22 patients with long PFS and 11 patients with short PFS. N=15 patients received ICI first-line (CHT-naive) and n=18 subsequent-line (CHT-treated) (**Table 1**). **Figure 1A** shows the study design in a flowchart.

### Human urine EV proteins correlate with PFS and biological pathways

For baseline analyses, the follow-up measurements on urine samples (n=17) were excluded. LC/MS analysis revealed 6183 proteins in urine EV samples. The proteins were derived from multiple species; thus, by utilizing the NCBI Taxonomy database, we divided the proteins into four taxon-related categories: human (n=3513), bacterial (n=2647), fungal (n=19), and viral (n=4). Due to the low number of fungal and viral metabolites detected in our screen, we were not able to analyze proteins from these taxonomic units. First, we analyzed Spearman's correlation between the relative abundances of all sequenced human proteins in baseline urine samples and PFS in months (**Figure 1B**). Here, we found that multiple proteins show association with PFS (r(s)>|0.3|, p<0.05, n=191), including BPIFB2, F11R, WDR62, MPP5, FCGBP and TFF3 that a significant positive correlation; while TMEM176B, TNC, RAB4B, CD74, NOMO3, ARG1 and PINK1 had a significant negative correlation. Next, we performed WRS tests and ROC analyses for all urine protein proteins, comparing patients according to short-term PFS (≤6 months) and long-term PFS (>6 months), PD-L1 IHC expression [high (>50%) vs low ≤50%)] and the line of ICI (CHT-naive vs CHT-treated). From the 3513 human proteins, 186 showed differential abundance (p<0.05) according to PFS groups, with 101 being significantly more abundant in patients with short PFS and 85 in patients with long PFS.

For the interpretation of biological pathways, we used Over-Representation Analysis (ORA) and the Reactome and GO biological processes databases (**Figures 1C, D**). Affinity propagation was utilized to eliminate redundant pathways. **Supplementary Figure 1** shows all pathway results without filtering algorithms. Human urinary EV proteins associated with long PFS constituted pathways in connection with general immune function and innate immunity. However, proteins associated with short PFS contributed to highly specific pathways, including the Endosomal/Vacuolar pathway, Complement cascade, COPI-mediated anterograde transport (Reactome), Nucleobase-containing small molecule biosynthetic process, and nucleoside trisphosphate metabolic process (GO biological process). Neutrophil degranulation was present in the proteomic profiles of both patient groups, but a much higher enrichment with lower FDR was detected in patients with short PFS.

### Top differentially abundant human urine EV proteins in patients with short- and long-term progression-free survival

**Figure 2:**
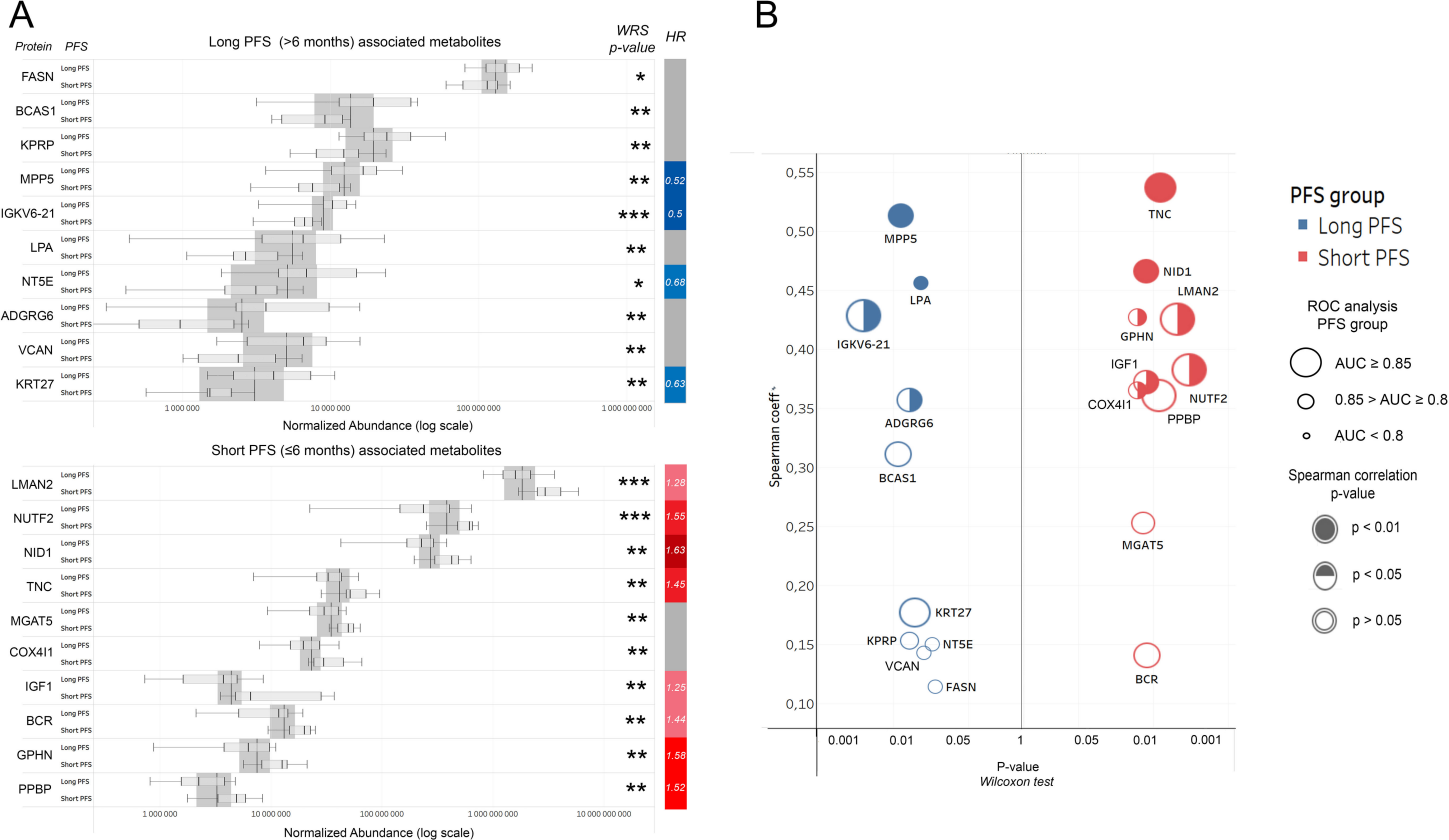
Top human urine EV proteins according to PFS. **(A)** Bar charts show relative abundances of the top 10 proteins associated with long or short PFS according to the Wilcoxon rank-sum (WRS) test. The y axis indicates proteins and their corresponding abundance levels in PFS groups, X-axis shows normalized abundance on a logarithmic scale. The vertical bar on the right displays the hazard ratio (HR) for proteins with a significant multivariate Cox regression (p<0.05) regarding PFS. **(B)** P-values generated by the WRS test (X-axis) for the top 10 long and short PFS-associated EV proteins plotted against their Spearman's correlation coefficient (Y-Axis), where the color code (blue vs. red) indicates the corresponding PFS group, circle size indicates AUC from corresponding ROC analysis, and circle filling the p-values for Spearman's correlations. *p < 0.05, **p < 0.01, ***p < 0.001.

We aimed to highlight the top 10 abundant metabolites according to PFS (**Figure 2A**). **Figure 2B** shows the top 10 abundant proteins in patients with short vs. long PFS plotted against Spearman's correlation coefficient and ROC AUC. Considering all 3 statistical measurements, MPP5, IGKV6-21, and ADGRG6 showed the strongest association with long PFS, while TNC, NID1, LMAN2, and NUTF2 with short PFS. Cox hazard regression was performed for top 10 abundant proteins in patients with short- and long PFS, where MPP5, IGKV6-21, NT5E and KRT27 were significant positive predictors of PFS, and LMAN2, NUTF2, NID1, TNC, IGF1, BCR, GPHN and PPBP were significant negative predictors of PFS (**Figure 2A**, **Table 2**). **Supplementary Figures 2A, B** show the top 10 abundant proteins in CHT-naive and CHT-treated patients, and PD-L1 high and PD-L1 low patients.

**Table 2:** Cox hazard regression for top 10 abundant human EV proteins in patients with long PFS and short PFS.

Covariate	b	SE	Wald	P	Exp(b)	95% CI of Exp(b)
LONG PFS						
**FASN**	-2,57E-01	2,39E-01	1,1546	0,2826	0,7737	0,4857 to 1,2325
**BCAS1**	-0,02044	0,0587	0,1212	0,7277	0,9798	0,8738 to 1,0986
**KPRP**	-0,1887	0,1503	1,578	0,2091	0,828	0,6177 to 1,1099
**MPP5**	-0,6451	0,2074	9,6722	**0,0019**	0,5246	0,3501 to 0,7861
**IGKV6_21**	-0,6841	0,238	8,2641	**0,004**	0,5046	0,3172 to 0,8025
**LPA**	-0,1042	0,1032	1,0205	0,3124	0,901	0,7368 to 1,1018
**NT5E**	-0,3733	0,1525	5,9924	**0,0144**	0,6885	0,5114 to 0,9269
**ADGRG6**	-0,6089	0,3298	3,4099	0,0648	0,5439	0,2859 to 1,0347
**VCAN**	-0,1765	0,1236	2,0394	0,1533	0,8382	0,6586 to 1,0666
**KRT27**	-0,4539	0,1805	6,3222	**0,0119**	0,6351	0,4467 to 0,9031
SHORT PFS						
**LMAN2**	0,2518	0,1064	5,6019	**0,0179**	1,2863	1,0453 to 1,5828
**NUTF2**	0,4416	0,1518	8,4623	**0,0036**	1,5552	1,1567 to 2,0910
**NID1**	0,4917	0,1997	6,065	**0,0138**	1,6351	1,1078 to 2,4134
**TNC**	0,375	0,1265	8,7832	**0,003**	1,455	1,1369 to 1,8622
**MGAT1**	-0,4245	0,2419	3,0807	0,0792	0,6541	0,4082 to 1,0482
**COX4I1**	0,1813	0,1145	2,5073	0,1133	1,1988	0,9589 to 1,4987
**IGF1**	0,04917	0,02275	4,6722	**0,0307**	1,2504	1,0048 to 1,4980
**BCR**	0,366	0,1363	7,2133	**0,0072**	1,442	1,1055 to 1,8810
**GPHN**	0,4636	0,1654	7,8578	**0,0051**	1,5898	1,1515 to 2,1949
**PPBP**	0,4236	0,1537	7,598	**0,0058**	1,5275	1,1320 to 2,0613

Bold: Significant at p<0.05.

To reveal whether the relative abundance of top PFS-related human proteins changed during IT, we compared baseline vs. follow-up urine samples on a sub-cohort of patients with long PFs (n=17, **Supplementary Figure 3**). Only the abundance levels of BCAS1 and KRT27 were significantly altered (decreased) in the follow-up samples compared to baseline. We could obtain follow-up samples from only 2 patients with short PFS, so we could not perform further statistical analysis for this group.

### Gut microbial signatures correlate with urine EV bacterial protein abundance

**Figure 3:**
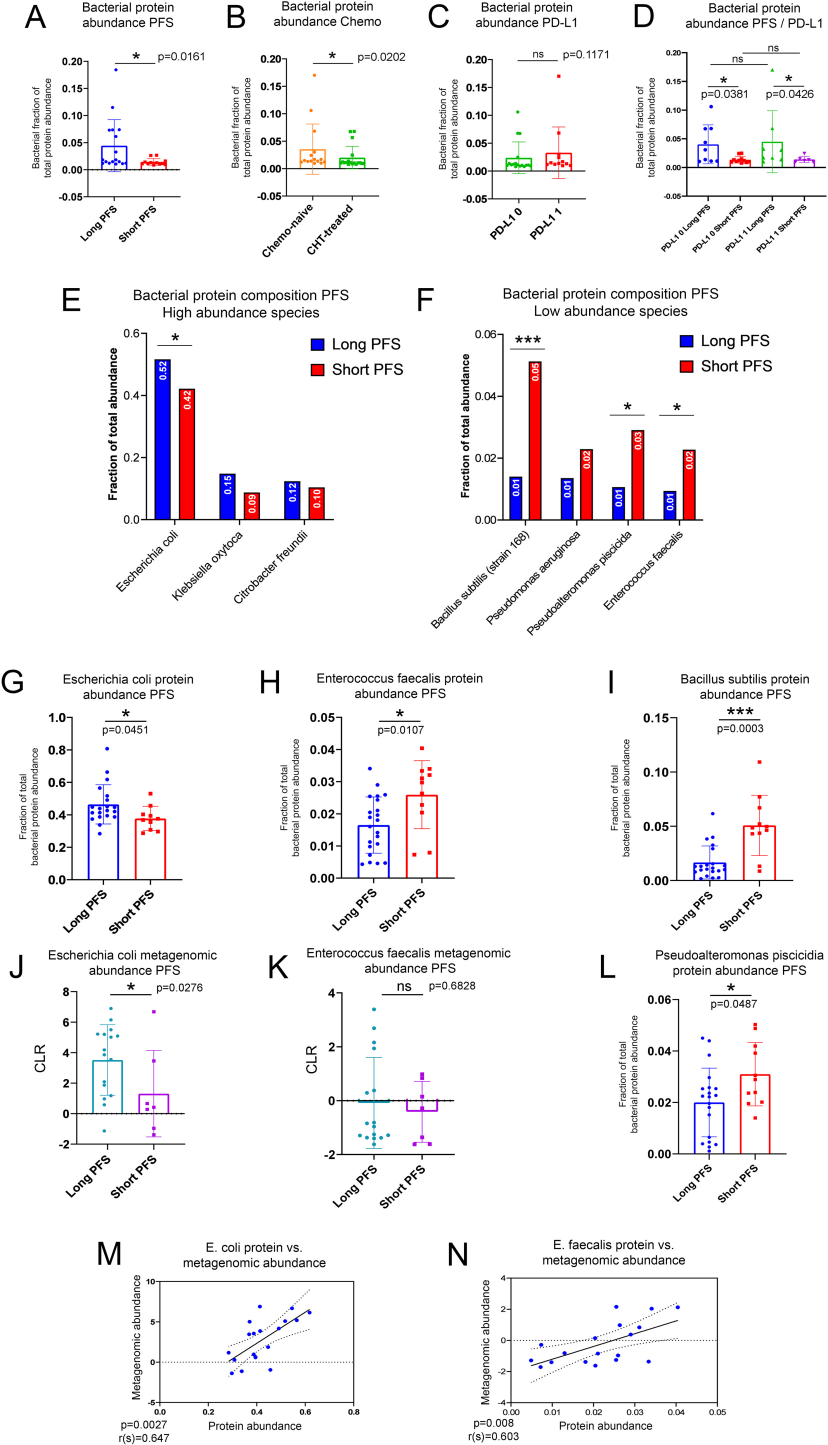
Characterization of the urine EV bacterial proteome in the context of the gut microbiome. Bar charts show the bacterial fraction of total EV protein abundance in urine samples comparing patients with short- and long PFS **(A)**, CHT-naive with CHT-treated patients **(B)**, and patients with high vs low PD-L1 IHC expression **(C)**. A significantly higher fraction of the urine EV proteome was of bacterial origin in patients with long PFS vs short PFS [p=0.0161, **(B)**] and in CHT-naive patients vs CHT-treated [p=0.202, **(B)**]. The increased fraction of bacterial urine EV proteins in patients with long PFS were also present in PD-L1-low (p=0.0381) and high (p=0.0426) subgroups **(D)**. There was no significant difference between PD-L1-low and PD-L1-high patients [p=0.1171, **(C)**]. The highest fraction of bacterial urine EV proteins was of *Escherichia coli* origin [49.3%, **(E)**], with a significantly higher fraction in patients with long vs short PFS [p=0.0451, **(G)**]. Two more species were represented above 10% of the total bacterial proteome: *Klebsiella oxytoca* and *Citrobacter freundii*, but none of them exhibited significant difference according to PFS **(E)**. *Bacillus subtilis* [p=0.0003, **(I)**], *Pseudoalteromonas piscicida* [p=0.0487, **(L)**] and *Enterococcus faecalis* [p=0.0107, **(H)**] exhibited significantly increased fraction of their bacterial proteome in patients with short PFS (compared to long PFS), but all contributed to the total bacterial urine EV proteome below 10% **(F)**. The gut metagenomic abundance of *E. coli* significantly differed in patients with long vs. short PFS [p=0.0276, **(J)**], but not in the case of *E*. *faecalis* [p=0.6828, **(K)**]. Still, the gut metagenomic abundance of both *E. coli* (r=0.647, p=0.0027) and *E*. *faecalis* (r=0.603, p=0.008) showed a strong significant positive correlation with its corresponding urine protein EV abundance **(M, N)**. *p < 0.05, ***p < 0.001.

A total of n=2647 bacterial proteins were detected in urine EV samples annotated taxonomically with NCBI databases. The abundance fraction of bacterial per total urine EV proteins is significantly higher in patients with long PFS (vs. short PFS, **Figure 3A**) and in CHT-naive (vs. CHT-treated, (**Figure 3B**) patients, however, there is no significant difference according to PD-L1 expression (**Figure 3C**). A significant difference between patients with short and long PFS was similarly detectable in PD-L1 low and PD-L1 high subgroups (**Figure 3D**). These results suggest that patients with long PFS exhibit a higher amount of circulating bacterial components that can be detected from urine samples.

Shotgun metagenomics was performed on stool samples of a sub-cohort of n=23 patients (16 with long PFS and 7 with short PFS) to correlate the bacterial signature of the gut with the urine EV bacterial proteome. In the gut microbiome, phyla *Firmicutes, Actinobacteria*, and *Spirochetes* were overrepresented in patients with short PFS, and *Verrucomicrobia* was overrepresented in patients with long PFS (**Supplementary Figure 4**). Genera *Akkermansia, Bacteroides, Barnesiella, Escherichia, Parabacteroides*, and *Paraprevotella* were overrepresented in patients with long PFS, and *Bifidobacteria* and *Streptococcus* were overrepresented in patients with short PFS (**Supplementary Figure 4**). Previously, a more comprehensive metagenomic analysis of a sizeable patient cohort (n=62) showed similar findings regarding long and short PFS-associated key bacterial taxa (30)

Proteins in the urinary EV bacterial proteome were associated with taxa according to their relative abundance. The majority of identified proteins derive from *Escherichia coli* (49%), *Klebsiella oxytoca* (13%), and *Citrobacter freundii* (12%), from which *E. coli* proteins were significantly more abundant in patients with long PFS (compared to short PFS, **Figure 3E**). We detected 4 more taxa with identified urine proteins above 1% of total bacterial protein abundance: *Bacillus subtilis, Pseudomonas aeruginosa, Pseudoalteromonas piscicida* and *Enterococcus faecalis*. Urine EV proteins for *B. subtillis, P. piscicida*, and *E. faecalis* were significantly more abundant in patients with short PFS compared to long PFS (**Figure 3F**). The abundance of urine EV proteins was assessed according to their fraction of total bacterial EV protein abundance (**Figures 3G–I, L**). We also evaluated the taxonomical origin of bacterial urine proteins concerning phyla and classes. We found that *Proteobacteria*- and *Firmicutes-* derived proteins are significantly more abundant in patients with long PFS (p<0.001, and p=0.001, respectively) compared to patients with short PFS (**Supplementary Figure 5A**). At class level, *Gammaproteobacteria* (p<0.001), *Desulfitobacteriia* (p=0.016), and *Bacilli* (p=0.005) showed significantly increased abundance in patients with long PFS compared to short PFS (**Supplementary Figure 5B**)

When evaluating gut metagenomic abundance of the same taxa, we revealed that abundance of *E. coli* and *E. faecalis* were similarly increased in patients with long PFS compared to short PFS (**Figures 3J, K**). Furthermore, the gut metagenomic abundance and urine EV protein abundance of these two species both showed significant positive correlation. This suggests that bacterial urine proteins associated with these taxa originated from the gut microbiome (**Figures 3M, N**). *B. subtilis* and *P. piscicida* were not detected in the metagenomic data, so their urinary EV proteins might originate from a biological compartment other than the gut microbiome.

### The bacterial urine EV proteome in the context of progression-free survival

**Figure 4:**
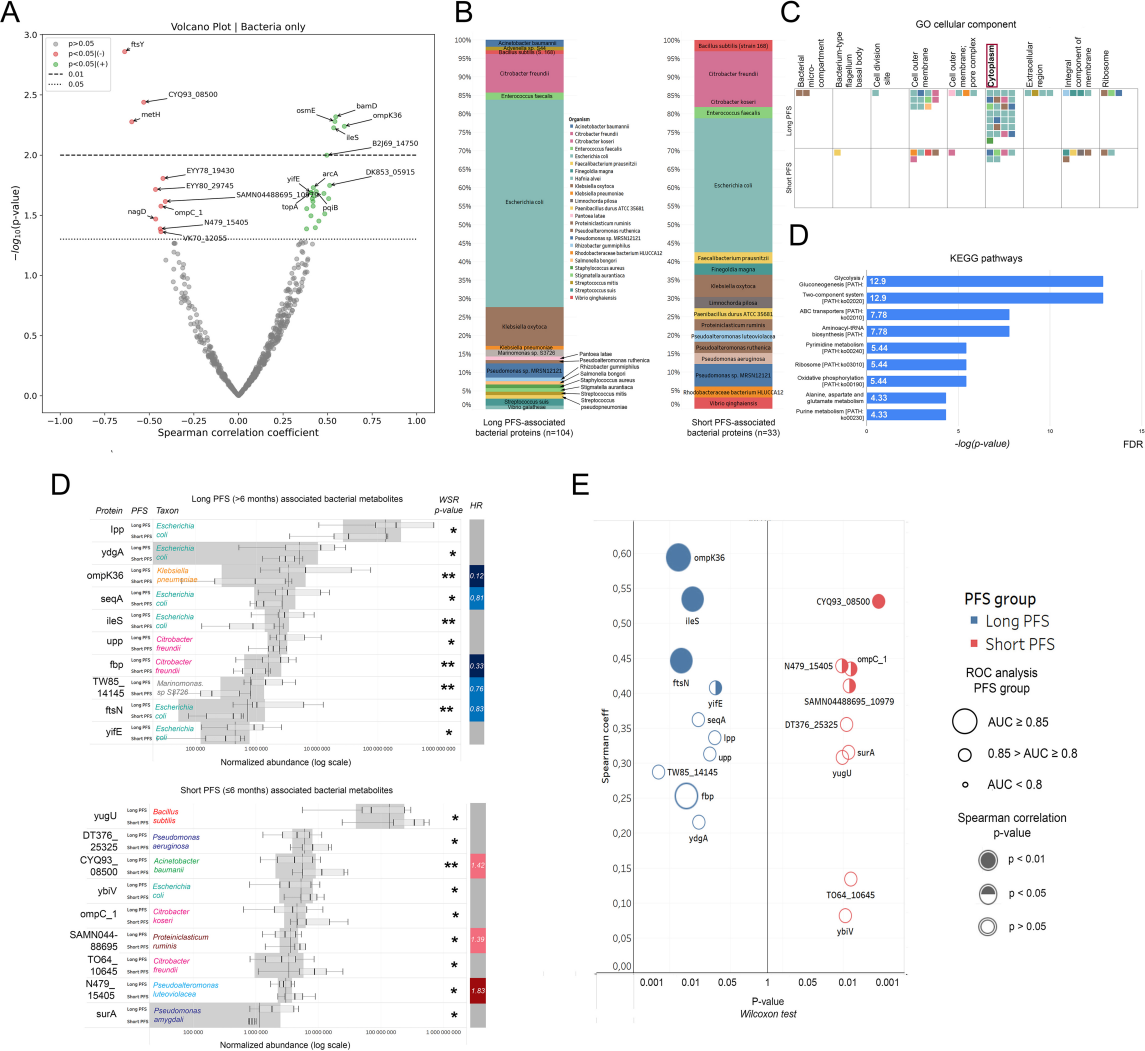
Correlation of the bacterial urine EV proteome with PFS, taxonomy, and molecular function. Top bacterial urine EV proteins according to PFS. **(A)** The volcano plot displays bacterial urine EV proteins according to their correlation with PFS in months. Spearman's correlation coefficient is shown on the X-axis and the corresponding -*log*10 (p-value) on the Y-axis. Non-significant bacterial proteins are grey; those showing significant positive correlation (p<0.05) with PFS are green, and those showing significant negative correlation are red. The dotted line indicates p<0.05-, the dashed line indicates p<0.01 threshold. **(B)** Stacked bar charts show the phylogenetic composition of long (n=104) vs short (n=33) PFS-associated bacterial EV proteins. **(C)** Using these 137 proteins, pathway analysis was carried out with the FUNAGE-Pro pipeline. The GO cellular component database was used to determine that cytoplasmic proteins (both long and short PFS-associated) were more enriched in the urine EV proteome than proteins from other compartments. Colorized squares represent individual proteins. Color coding for taxa is shown in panel **(B, D)**. Panel **(D)** shows enriched metabolic pathways from long PFS-associated proteins according to the KEGG database. The X-axis represents [-log]p-value with Benjamini–Hochberg multiple testing correction. **(E)** Bar charts show relative abundances of the top 10 and 9 proteins associated with long or short PFS according to Wilcoxon rank-sum (WRS) test (There were only 9 bacterial proteins with a significant WRS p-value differentially abundant in patients with short PFS). The Y-axis indicates proteins and their corresponding abundance in PFS groups; X-axis shows normalized abundance on a logarithmic scale. The vertical bar on the right displays the hazard ratio (HR) for proteins with a significant multivariate Cox regression (p<0.05) regarding PFS. **(F)** P-values generated by the WRS test (X-axis) for the top 10 long and short PFS-associated bacterial proteins plotted against their Spearman's correlation coefficient (Y-axis) are shown in panel **(E)**, where color code (blue vs. red) indicates the corresponding PFS group, circle size indicates AUC from corresponding ROC analysis, and circle filling the p-values for Spearman's correlations. *p < 0.05, **p < 0.01.

Spearman's rank correlation was used for the association of relative abundances of all sequenced bacterial proteins in baseline urine EV samples and PFS in months (**Figure 4A**). We found that multiple bacterial proteins showed association with PFS (r(s)>|0.3|, p<0.05, n=31), including ompK36 (Outer membrane porin, analogous to *E. coli* OmpF), ileS (Isoleucine-tRNA ligase), osmE (Osmotically-inducible putative lipoprotein), bamD (Outer membrane protein assembly factor) and yifE (UPF0438 protein) had a significant positive correlation; while ftsY (Signal recognition particle receptor), CYQ93_08500 (Beta-lactamase of *Acinetobacter baumannii*), metH (Methionine synthase), EYY78_19430 (Cyclic diguanylate phosphodiesterase) and omp_C1 (outer membrane porin C) had a significant negative correlation. WRS test demonstrated that from the 2647 bacterial proteins, 96 showed differential abundance (p<0.05). According to PFS groups, only 19 showed significantly increased abundance in patients with short PFS and 77 in patients with long PFS. Based on Spearman’s correlation and differential expression, we identified a total of 137 PFS-associated bacterial proteins.

**Figure 4B** shows the phylogenetic origin of PFS-associated bacterial proteins in urine EV samples. 63% of long PFS-associated proteins and 36% of short PFS-associated proteins were derived from *E. coli*. Gene enrichment analysis using the FUNAGE-Pro functional analysis pipeline (47) was used to reveal gene enrichment according to cellular compartments (GO) and biological pathways (KEGG). We found that cytoplasmic bacterial proteins were significantly overrepresented compared to proteins from other prokaryotic cell compartments (p=0.008, **Figure 4C**). Pathway analysis using the KEGG database revealed that none of the pathways were significantly enriched from short PFS-associated proteins. In contrast, multiple pathways showed significant enrichment from long PFS-associated proteins, including Glycolysis/Gluconeogenesis (p<0.001), Two-component system (p<0.001), ABC transporters (p=0.004) and Aminoacyl-tRNA synthesis (p=0.004) among others (**Figure 4D**).

The top 10 abundant bacterial urine EV proteins are displayed in every patient group according to PFS (**Figure 4E**), the line of ICI (**Supplementary Figure 2C**) and PD-L1 IHC expression (**Supplementary Figure 2D**). While the majority of long PFS-associated bacterial proteins showed an origin of *E. coli*, short PFS-associated proteins originated from multiple different taxonomic groups, ybiv (Cof-type HAD-IIB family hydrolase) being the only *E. coli* protein (**Figure 4E**). **Figure 4F** shows the top 10 abundant EV proteins in patients with short vs. long PFS plotted against Spearman's correlation coefficient and ROC AUC. OmpK36, IleS, and ftsN (Cell division protein of *E. coli*) showed the strongest association with long PFS and CYQ93_08500, N479_15405 (Uncharacterized protein from *Pseudoalteromonas luteoviolacea*), omp_C1, and SAMN04488695 (Gram positive anchoring domain-containing protein) with short PFS, derived from the 3 statistical analyses. Moreover, multivariate Cox hazard regression showed that ompK36, seqA, fbp (Fructose-1,6-bisphosphatase class 1), TW85_14145 (Histidine kinase from *Marinomonas* sp. *S3726*) and ftsnN were significant independent predictors of long PFS, whereas CYQ93_08500, SAMN04488695 and N479_15405 were significant independent predictors of short PFS (**Figure 4E**, **Table 3**).

**Table 3:** Cox hazard regression for top 10 (Long PFS) and 9 (Short PS) abundant bacterial EV proteins in patients with long PFS and short PFS.

Covariate	Bacterial Species	b	SE	Wald	P	Exp(b)	95% CI of Exp(b)
LONG PFS							
**lpp**	*Escherichia coli*	-0.249	0.1496	2.7702	0.096	0.7796	0.5823 to 1.0437
**ydgA**	*Escherichia coli*	-0.4966	0.2737	3.2916	0.0696	0.6086	0.3569 to 1.0378
**ompK36**	*Klebsiella pneumoniae*	-2.0643	0.9133	5.1084	**0.0238**	0.1269	0.0214 to 0.7533
**seqA**	*Escherichia coli*	-0.2007	0.08705	5.3139	**0.0212**	0.8182	0.6904 to 0.9696
**ileS**	*Escherichia coli*	-0.3214	0.1833	3.0744	0.0795	0.7251	0.5072 to 1.0367
**upp**	*Citrobacter freundii*	-0.1001	0.1133	0.7804	0.377	0.9047	0.7254 to 1.1285
**fbp**	*Citrobacter freundii*	-1.0897	0.4679	5.4237	**0.0199**	0.3363	0.1350 to 0.8375
**TW85_14145**	*Marinomonas sp. S8726*	-0.2721	0.1273	4.5685	**0.0326**	0.7618	0.5944 to 0.9764
**ftsN**	*Escherichia coli*	-0.1758	0.0841	4.3693	**0.0366**	0.8388	0.7119 to 0.9883
**yifE**	*Escherichia coli*	-0.2967	0.1628	3.3189	0.0685	0.7433	0.5411 to 1.0211
SHORT PFS							
**yugU**	*Bacillus subtilis*	0.05441	0.04942	1.2122	0.2709	1.0559	0.9589 to 1.1627
**DT376_25325**	*Pseudomonas aeruginosa*	0.1759	0.1207	2.1262	0.1448	1.1924	0.9424 to 1.5087
**CYQ93_08500**	*Acinetobacter baumanii*	0.3515	0.1353	6.7466	**0.0094**	1.4212	1.0916 to 1.8503
**ybiV**	*Escherichia coli*	0.1595	0.09888	2.601	0.1068	1.1729	0.9672 to 1.4223
**ompC_1**	*Citrobacter koseri*	-0.04425	0.03773	1.3753	0.2409	0.9567	0.8888 to 1.0298
**SAMN04488695_10979**	*Proteiniclasticum ruminis*	0.3302	0.1291	6.5436	**0.0105**	1.3912	1.0816 to 1.7893
**TO64_10645**	*Citrobacter freundii*	0.02446	0.1498	0.02665	0.8703	1.0248	0.7651 to 1.3725
**N479_15405**	*Pseudoalteromonas luteoviolaceae*	0.605	0.3082	3.853	**0.0497**	1.8313	1.0040 to 3.3404
**surA**	*Pseudomonas amygdali*	0.08778	0.3437	0.06523	0.7984	1.0918	0.5585 to 2.1341

Bold: Significant at p<0.05.

We compared baseline vs follow-up urine EV samples in the context of the bacterial proteome. From long PFS-associated proteins, only the abundance of seqA increased significantly in follow-up samples. In contrast, from short PFS-associated proteins, the abundance of DT376_25325 (Uncharacterized protein from *Pseudomonas aeruginosa*), surA (Chaperone SurA from *Pseudomonas amygdali*), and TO64_10645 (DeoR family transcriptional regulator from *Citrobacter freundii*) showed a significant increase, and ompC_1 showed a significant decrease in follow-up samples compared to baseline (**Supplementary Figure 6**).

### Principal component analysis and machine learning approach

**Figure 5:**
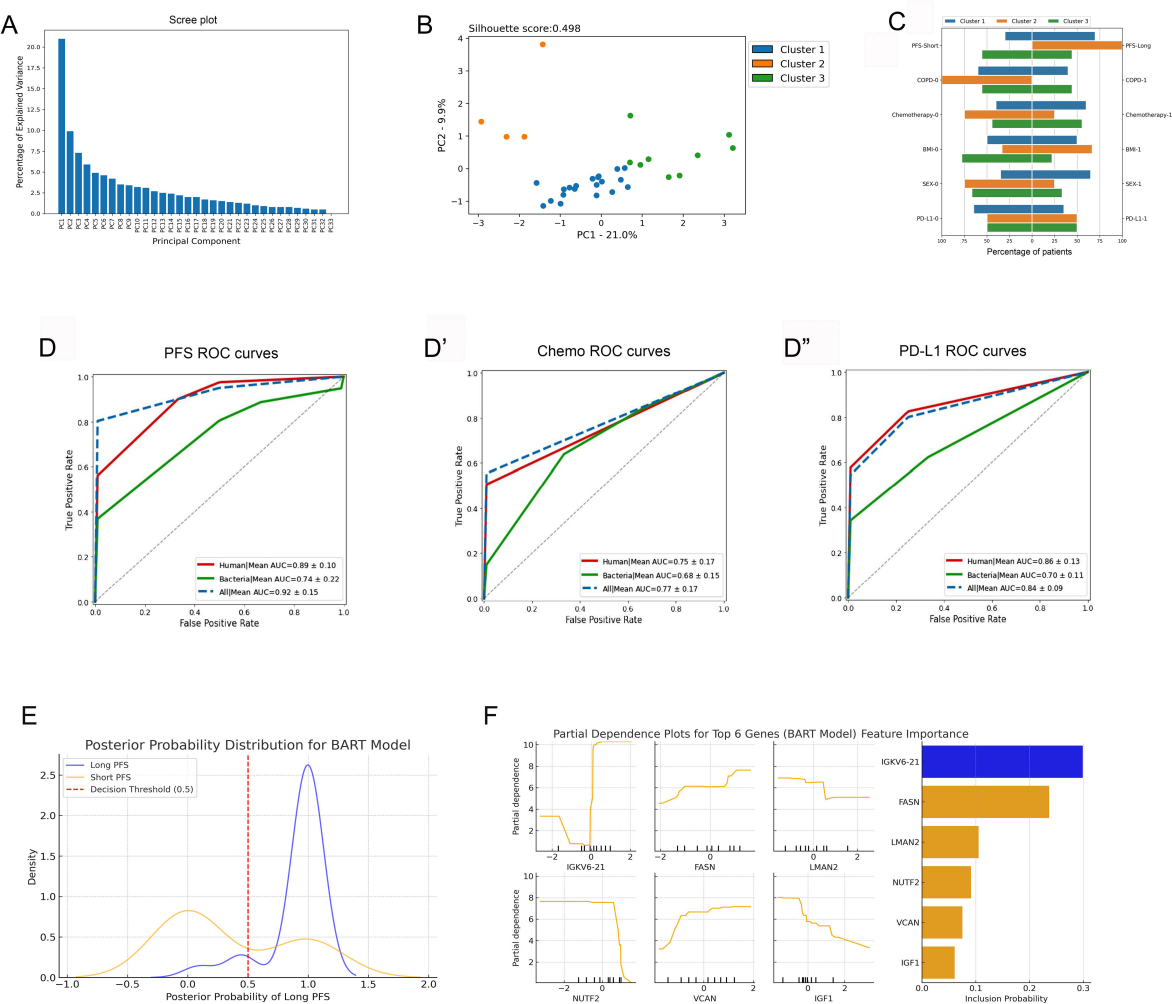
PCA and machine learning approach. **(A)** Principal component analysis (PCA), including PFS-associated human and bacterial EV proteins, revealed 3 main patient clusters **(A–C)**. The scree plot shows identified principal components (PCs) and their contribution in explaining the variance in our data. The first two PCs were utilized for clustering patients with the K-means clustering algorithm, generating 3 main clusters [silhouette score: 0.498, **(B)**]. **(C)** Panel **C** shows the clinicopathological characteristics of the 3 patient clusters. **(D-D”)** Results of the Random Forest (RF) machine learning algorithm using 5-fold cross-validation are shown in panels. In all settings, the top 20 differentially abundant human or bacterial proteins were used to run the training and validation datasets. In predicting short vs long PFS, top human proteins performed superiorly (AUC=0.89, F1 = 0.93, Accuracy=95%), top bacterial proteins performed fairly (AUC=0.74, F1 = 0.85, Accuracy=91%), and using both the top human and bacterial proteins yielded an outstanding result (AUC=0.93, F1 = 0.95, Accuracy=95%). Human and bacterial EV protein profiles predicted CHT status with an AUC of 0.75 (F1 = 0.74, Accuracy=70%) and with an AUC of 0.68 (F1 = 0.73, Accuracy=77%), respectively. A combined dataset (human + bacterial proteins) reached an AUC of 0.77 (F1 = 0.78, Accuracy=75%). PD-L1 status (high vs low) was predicted with an AUC of 0.86 (F1 = 0.79, Accuracy=77%) using only the top human proteins and with an AUC of 0.7 (F1 = 0.56, Accuracy=47%) using only the top bacterial proteins. The combined dataset reached an AUC of 0.84 (F1 = 0.79, Accuracy=73%). **(E)** Posterior Probability Distribution Plot shows BART-derived probability estimates for Long (blue) and Short (orange) PFS groups. The X-axis represents the posterior probability of Long PFS, while the Y-axis indicates density. The red dashed line (0.5 threshold) separates predicted Long PFS patients (>0.5). Long PFS cases cluster near 1, while Short PFS cases are more spread out. **(F)** Partial Dependence Plots (left panel) illustrate how protein abundance (X-axis) influences the model-predicted probability of Long PFS (Y-axis), while the Feature importance plot (right panel) ranks genes by posterior inclusion probability (X-axis).

PCA and machine learning approach was used to establish an integrated model of human and bacterial urine EV protein profiles. First, PCA was performed merging human and bacterial proteins exhibiting significant correlation with PFS (r(s)>|0.3|, p<0.05, n=191), where k-means clustering revealed 3 main clusters, plotting the first two PCs (**Figures 5A, B**). **Supplementary Figure 7** shows human and bacterial urine EV proteins with the greatest variation among clusters. In Cluster 1, patients with long PFS, subsequent line ICI, low PD-L1 expression, and female gender were overrepresented, whereas Cluster 2 consisted of patients with long PFS and no COPD. Also, patients with first-line ICI-treatment and CHT-naivety were overrepresented in this cluster. Cluster 3 included the highest number of patients with short PFS, where male gender and high BMI (>30 kg/m^2^) were also overrepresented (**Figure 5C**).

Random Forest (RF) machine learning approach was used with 5-fold cross-validation to verify the relevance and robustness of our predictive human and bacterial EV proteome profile in the context of PFS, chemotherapy, and PD-L1 expression. The top 20 human urine proteins gave a performance with an AUC of 0.89 and an accuracy of 95% when predicting short vs long PFS. Top bacterial proteins performed fairly, but were inferior to human proteins with an AUC of 0.74 and an accuracy of 91%. When combining both proteome signatures, the model reached an outstanding AUC of 0.93 with an accuracy of 95% (**Figure 5D**). Front-line CHT status (CHT-treated vs CHT-naive) was predicted with only moderate performance using either human (AUC=0.75) or bacterial proteins (AUC=0.68) (**Figure 5D’**). The model performed well in predicting PD-L1 status (high vs low) when using human proteins (AUC=0.86), but only moderately when using bacterial proteins (AUC=0.7) (**Figure 5D”**). **Supplementary Figure 8** shows associated confusion matrices for the 5-fold cross validation and the RF algorithm for every tested binary parameter.

Bayesian Additive Regression Trees (BART), an ensemble model, was selected as an additional non-linear Bayesian validation method for the RF model to ensure robustness and consistency in classification performance. BART, unlike RF, incorporates posterior probability distributions, enabling probabilistic classification and uncertainty estimation. We applied BART to capture non-linear protein interactions affecting PFS classification and to validate RF’s predictive performance in a Bayesian framework. This approach helps assess reproducibility and mitigates overfitting risks in a small dataset (n=33). BART supports RF’s findings with 78.6% accuracy and an AUC-ROC of 0.84. Its high recall (86.0%) indicates strong sensitivity in identifying Long PFS patients, while precision (84.7%) suggests some misclassification in borderline cases. Despite this, BART confirms the human 20-protein signature’s predictive power, reinforcing RF’s results. Furthermore, BART provides posterior probability estimates, unlike RF, which only gives deterministic classifications. **Figure 5E** shows the posterior probability distribution for the model, where the Long PFS group clusters near 1, showing high classification confidence, while the Short PFS group is more dispersed, indicating greater uncertainty but minimal overlap, suggesting strong model discrimination. According to model’s feature importance analysis, IGKV6-21 was the strongest discriminator, frequently included in BART, while FASN, LMAN2, NUTF2, VCAN and IGF1 also show non-linear effects. The dependence plots suggest threshold-driven relationships, where specific abundance levels markedly impact prediction rather than following a simple linear trend (**Figure 5F**).

## Discussion

ICI is the standard of care therapy in NSCLC. Still, only about 20-30% of patients experience durable benefit from ICI treatment. A noninvasive assessment of circulating biomarkers such as urinary EV proteins is an innovative approach to identifying ICI-related prognostic and predictive biomarkers. In the current study, we analyzed the baseline urine EV proteome of 33 ICI-treated patients and identified n=3513 human and n=2647 bacterial proteins. 186 human- and 96 bacterial proteins showed differential abundance (p<0.05) according to PFS. Our analyses revealed that an increased bacterial to human urine EV protein ratio and an increased *E. coli* protein ratio (to total bacterial protein abundance) is associated with long PFS. We also included a subgroup of 23 patients with fecal metagenomic analysis and found that specific gut bacteria were correlated with urine fractions of related proteins. Using multivariate testing, we identified the most important host and bacterial urinary EV proteins showing the strongest association with long or short PFS and examined whether their abundance changes during the course of ICI treatment on a sub-cohort of 17 long-term survivor patients.

Gene enrichment and pathway analysis of the host urine EV proteome revealed significant enrichment of the Endosomal/Vacuolar pathway, Complement cascade, and COPI-mediated anterograde transport in Short PFS patients, based on Reactome database. The complement system is implicated in NSCLC progression, with anaphylatoxin receptor signaling and membrane attack complex formation promoting tumor development and metastasis (48–50). Complement proteins may serve as predictive plasma biomarkers (51). Dysregulation of the endosomal recycling pathway is a known hallmark of cancer progression (52). COPI-mediated transport, crucial for ER-Golgi trafficking, is highly conserved and associated with tumorigenesis (53, 54).Regarding GO database pathways, the upregulation of nucleobase-containing small molecule biosynthetic processes and nucleoside trisphosphate metabolic processes is expected and suggests that a high rate of tumor cell turnover may imprint on the urinary EV proteome, particularly in the context of aggressive malignant proliferation. The same GO pathways were found to be enriched in hepatocellular carcinoma (55) and breast cancer (56). Interestingly, the interpretation of metabolic pathways of nucleoside-synthesis is not limited to cancer cell proliferation but might also have implications for cancer immunity that can be exploited to improve immunotherapies (57).

Multivariate Cox-regression showed that human urine EV proteins MPP5, IGKV6-21, NT5E and KRT27 had the strongest association with long PFS; while LMAN2, NUTF2, NID1, TNC, IGF1, BCR, GPHN and PPBP had the strongest association with short PFS. Immunoglobulin Kappa Variable 6-21 (IGKV6-21) is the V region of the variable domain of immunoglobulin light chains that participate in antigen recognition produced by plasma cells. It has been described in a hemato-oncological setting in light-chain myelomas (58), while in solid tumors its elevated expression was only reported in necrotic endometrial tumors (59). The prominence of IGKV6-21 in the Long PFS group could indicate an active humoral immune response, potentially contributing to improved outcomes. In contrast, NT5E, or CD73, has been widely implicated in cancer as an adenosine-generating immune checkpoint (60), expressed on cancer-associated fibroblasts with a controversial role: a negative prognostic factor in head and neck carcinoma (61) and colorectal cancer (60), but a positive prognostic factor in lung and gastric cancers (62). KRT27 contributes to the structural integrity of epithelial cells through the assembly of keratin intermediate filaments, while MPP5 plays a key role in maintaining cell polarity. To date, these proteins they have not been associated explicitly to ICI-response in any malignancies, however MPP5’s role was recently demonstrated in liver cancer as a potential tumor suppressor (63).

L-type lectin LMAN2 impedes exosomal release in the exosomal-Golgi pathway (64) and is linked to unfavorable prognosis in HER2+ breast cancer (61). NUTF2, a GDP-binding protein, is involved in nucleocytoplasmic transport and correlates with cell proliferation, EMT markers, and upregulation in multiple cancers (65). NID1, another EMT marker, is essential for metastasis and chemoresistance in ovarian cancer and claudin-low cancers (66, 67). TNC promotes angiogenesis, invasion, metastasis, and T-cell immobilization in tumors (68, 69). Also, TNC was reported as a negative prognostic factor in lung cancer, regulating EMT, intratumoral immunosuppression, and was a plasma biomarker for pancreatic cancer but unreported in urine (70, 71). Elevated IGF1 is linked to increased risk of thyroid, colorectal, breast, prostate, and lung cancer (72–74). BCR, an ABL1 fusion partner in CML, is altered in lung adenocarcinoma and other cancers (75, https://www.aacr.org/professionals/research/aacr-project-genie/). GPHN is associated with chromosomal instability in colon cancer, but no lung cancer-related studies exist (76). PPBP and its chemokine CXCL7 influence tumor biology via CXCR1/2 binding and were proposed as early lung and gastric cancer biomarkers (57, 77, 78). Brocco et al. (28) similarly studied ICI-treated NSCLC patients, where they identified multiple plasma-derived EV proteins that overlap with our Short-PFS-related EV protein signature, including TNC, NID1, IGF1, and PPBP, highlighting the potential of urinary EV protein as a viable, non-invasive alternative for biomarker discovery in NSCLC immunotherapy response, that might facilitate home-based monitoring of ICI response, should suitable point-of-care diagnostic technologies be developed.

Microbiota generates a variety of peptides, proteins, and metabolites that influence host health and pathophysiological functions. Moreover, pathogen-associated molecular patterns (PAMPs) are unique to microbes critical for immune cell activation. (79). Protein components of microbiota can also pass through the gut barrier and enter circulation. To date, the urine proteome of bacterial origin was only studied in the context of urinary infections, renal diseases, and urogenital cancers (80, 81), but not in the context of non-urinary cancers. We revealed that an increased bacterial/host protein ratio in the urine EVs is more frequent in patients with long PFS and that the abundance of *E. coli* and *E. faecalis* proteins in the urine EVs positively correlates with PFS and their gut metagenomic abundance. The association between *E. coli* proteins in urinary EVs and improved PFS in NSCLC aligns with prior findings that *E. coli*, even intratumorally, predicts better ICI outcomes (82), possibly due to immune modulation or microbial adjuvanticity. Notably, whether *E. coli* translocation is a driver or a consequence of heightened ICI efficacy remains unresolved (83), warranting further investigation into its mechanistic role in lung cancer immunotherapy. The fact that purely the increased presence of bacterial proteins in the urine extracellular vesicles is predictive to immunotherapy response is intriguing and might be associated with gut permeability that depends on multiple factors, including genetic and environmental (84). Bacterial gene enrichment analysis showed that cytoplasmic bacterial proteins are overrepresented over the proteins from other prokaryotic compartments. This means that bacterial EVs not only contain outer membrane proteins but also cytosolic components, likely incorporated through passive entrapment during vesicle formation or selective sorting. These cytosolic proteins may play roles in quorum sensing, biofilm formation, and microbial communication, highlighting the functional complexity of bacterial EV cargos (85). Furthermore, bacterial pathways such as Glycolysis/Gluconeogenesis, Two-component system, ABC transporters, and Aminoacyl-tRNA synthesis are significantly overrepresented in patients with long PFS. However, no characteristic bacterial pathways were identified based on short PFS-associated proteins. Among the most reliable predictors of long PFS, Porin proteins ompk36 from *K. pneumoniae* and Omp_C1 from *E. coli* were identified as key structures in antibiotic resistance (86, 87), while seqA was shown to be crucial in *E. coli* DNA replication. Fructose-1,6-bisphosphatase class 1 (Fbp) and ftsN are all essential components of bacterial metabolism and cell wall synthesis (88, 89). To date, none of these proteins have been identified as potential plasma or urine biomarkers in cancer. Of note, due to the incompleteness and redundancy of bacterial protein data, certainty of several protein matches are never 100%. Moreover, given that Gram-negative bacteria actively shed outer membrane vesicles, their proteins may be overrepresented in urinary EVs, whereas Gram-positive bacterial EVs could be underdetected due to structural constraints, highlighting a potential bias in microbial EV composition. Altogether, the presence of microbial EV proteins in urine suggests a potential link between the gut-lung microbiome (90) axis and ICI response, thus, we strongly recommend further experimental validation to ascertain the predictive power of the bacterial proteins identified in our study.

Random forest machine learning model supported the reliability of our key human urine EV proteins, with an outstanding performance (AUC=0.89) and accuracy (95%), while bacterial EV proteins performed fairly in predicting PFS (AUC=0.74). This may be explained by the fact that there was no solid bacterial proteomic signature for patients with short PFS. Of note, key human proteins also performed well in predicting PD-L1 status (high vs. low, AUC=0.86). To confirm the predictive power of our best-performing protein signature, namely human EV proteins predicting PFS, we added a non-linear Bayesian method tailored to our small dataset and small number of analyzed features included: BART further validated our findings with comparable performance metrics to our RF model.

Limitations of this study include the relatively small sample size, particularly in the exploratory follow-up cohort; however, the primary focus was on baseline urinary EV proteome differences between responders and non-responders, and the follow-up data serve only for hypothesis-generating. Also, we cannot prove causality in the case of altered host or bacterial urine EV proteome relative to immunotherapy response. Further limitation is the absence of a healthy control group, therefore urine EV role in a broader biological context could not be assessed in our study. Validity of multiparameter machine learning models is frequently limited by overfitting, which we mitigated with 5-fold cross validation and the inclusion of non-linear Bayesian model to confirm the predictive power of human EV proteins in PFS groups. Still, future studies with a greater sample size, independent validation cohort and experimental validation are needed to confirm our findings.

## Conclusion

With multivariate tests we established a host-derived protein profile that predicted ICI outcomes with an AUC of 0.89 and an accuracy of 95% using the Random Forest algorithm. We also showed that an increased ratio of bacterial proteins in the urine extracellular vesicles was associated with long PFS, including an increased ratio of *E. coli* proteins. Furthermore, we found strong correlations between urine EV protein abundance and gut metagenomic abundance in the case of multiple bacteria. To our knowledge, this is the first study to identify the clinically predictive urine proteome in NSCLC patients treated with anti-PD1 ICI.

## Data Availability

The mass spectrometry proteomics data have been deposited to the ProteomeXchange Consortium via the PRIDE (91) partner repository with the dataset identifier PXD062630 and 10.6019/PXD062630.
