# Supplementary material for: Host and bacterial urine proteomics might predict treatment outcomes for immunotherapy in advanced non-small cell lung cancer patients
Source: Front Immunol. 2025 Apr 14;16:1543817. doi: 10.3389/fimmu.2025.1543817 (PMC12035445; doi:10.3389/fimmu.2025.1543817)
Supplement: Supplementary file 1 [file DataSheet1.docx]

**SUPPLEMENTARY MATERIAL**

**SUPPLEMENTARY FIGURES**


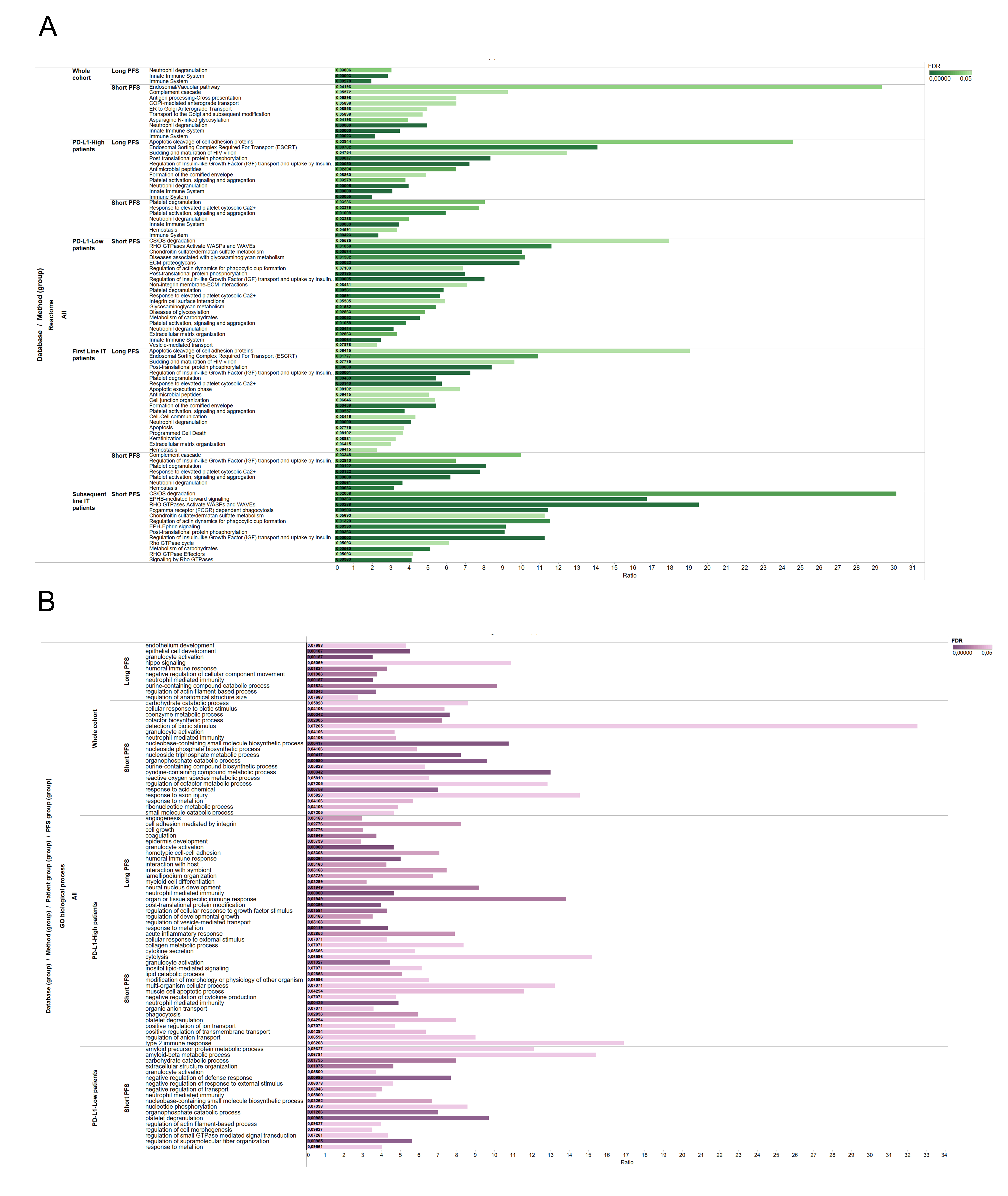


**SFig 1. Results of Enrichment and Pathway analyses without affinity propagation.** Pathway analyses using the Reactome (A) and GO biological process (B) databases were performed with ORA from whole exome datasets. Only proteins with significant correlation with PFS (in months) (r(s) > [0.3], p < 0.05), OR significant WRS test were included. Patients were stratified according to PFS in the whole cohort and also in PD-L1-low vs PD-L1-high and Chemo-naive (first line) vs Chemo-treated (subsequent line) subgroups. For multiple testing, Benjamini-Hochberg adjustment was used; False Discovery Rate (FDR) is indicated with color tone and labels in the horizontal bar charts. The enrichment ratio is displayed on the X-axis. FDR-values were considered significant with p<0.1. All pathways with a significant FDR value are indicated with no affinity propagation filtering used. In the case of the GO dataset, no PFS-associated processes with p<0.1 FDR were found if analyzed in Chemo-naive vs Chemo-treated subgroups.





**SFig 2. Top human- and bacterial urine proteins according to PD-L1 status and line of immunotherapy.** Bar charts show relative abundances of the top 10 human and bacterial urine proteins associated with Chemo-treated or Chemo-naive (A and C) and PD-L1 high- or PD-L1 low status (B and D) according to the Wilcoxon rank-sum (WRS) test. The y axis indicates protein metabolites and their corresponding abundance in PFS groups, X-axis shows normalized abundance on a logarithmic scale. **p < 0.05, **p < 0.01, ***p < 0.001*


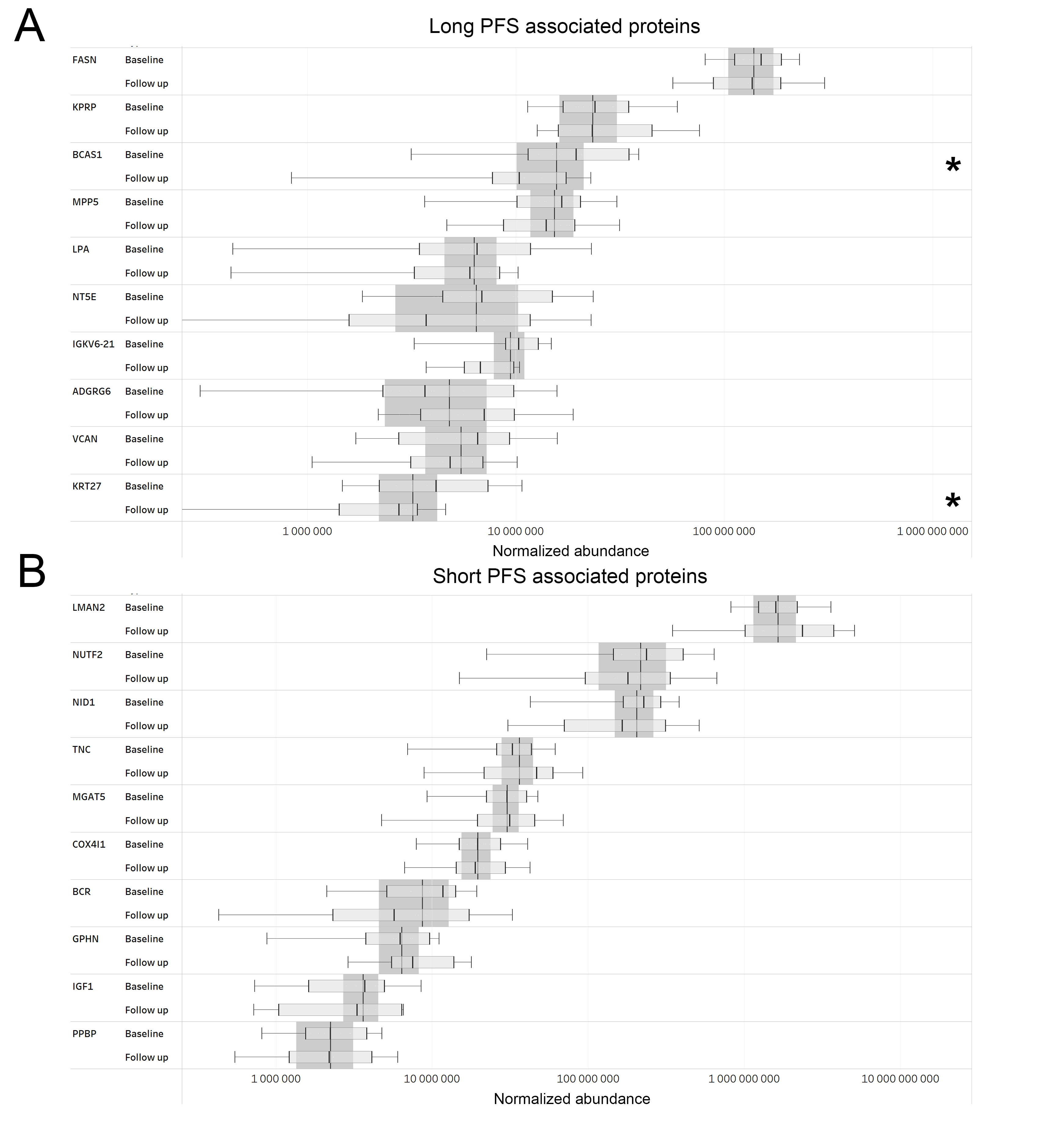


**SFig 3. Top human urine proteins: baseline vs follow up comparison.** Bar charts show relative abundances of the top 10 human urine proteins associated with long (A) and short (B) PFS relative to the time of sampling (baseline vs follow up). Statistical comparison is performed with the Wilcoxon rank-sum (WRS) test. **p < 0.05, **p < 0.01, ***p < 0.001*


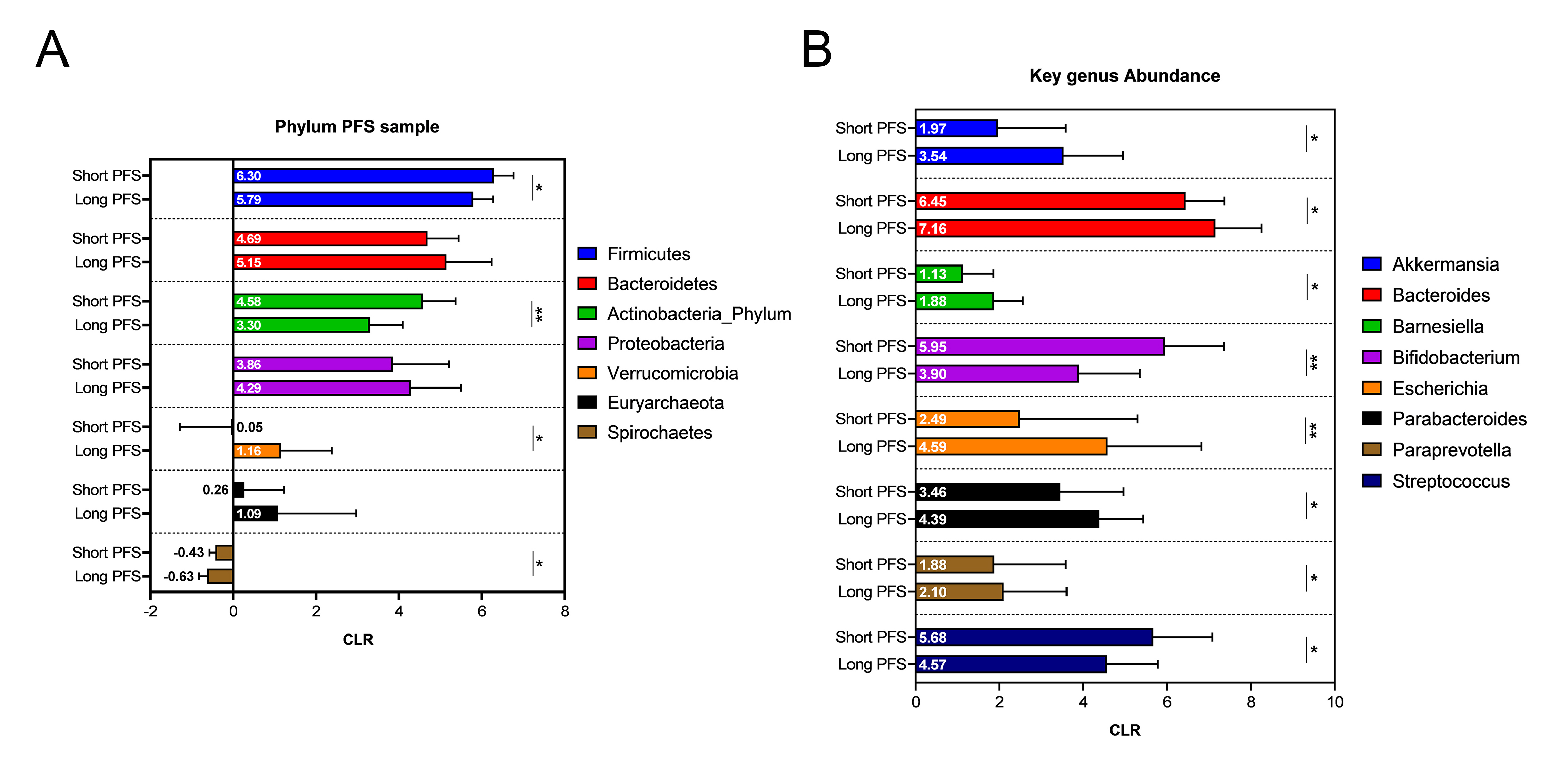


**SFig 4. Differentially abundant bacterial taxa in the gut microbiome.** Gut metagenome of n=23 patients (n=16 woth long PFS, n=7 with short PFS) has been analyzed. Relative abundance of bacterial phyla (A) and key genera (avCLR>1, B) are shown with bar charts according to long vs short PFS. X axis shows normalized CLR values. **p < 0.05, **p < 0.01, ***p < 0.001*


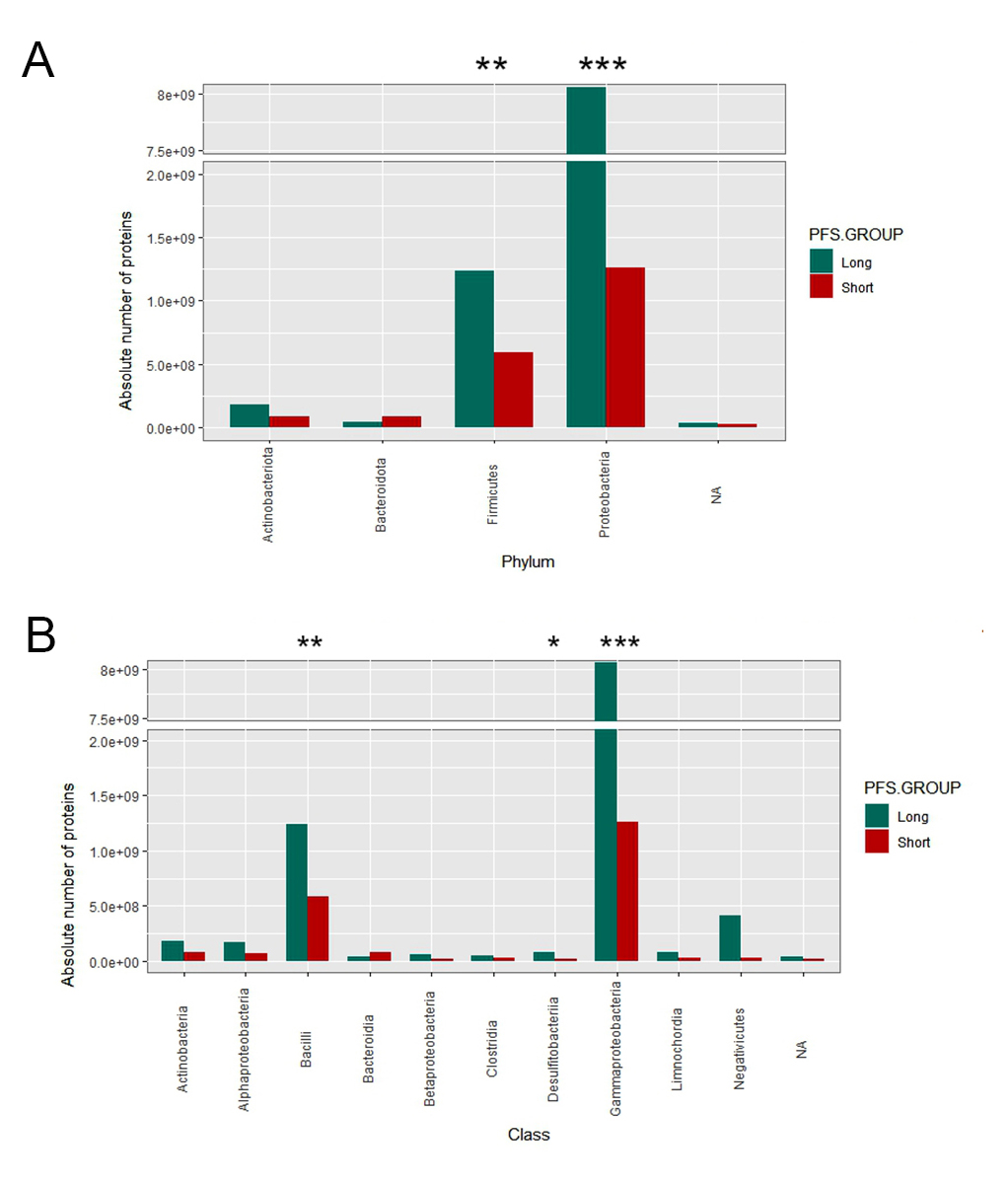


**SFig 5. Bacterial origin of urine proteins: phyla and classes.** Comparison between the absolute abundance of bacterial EV proteins according to phylum (A) and Class (B).


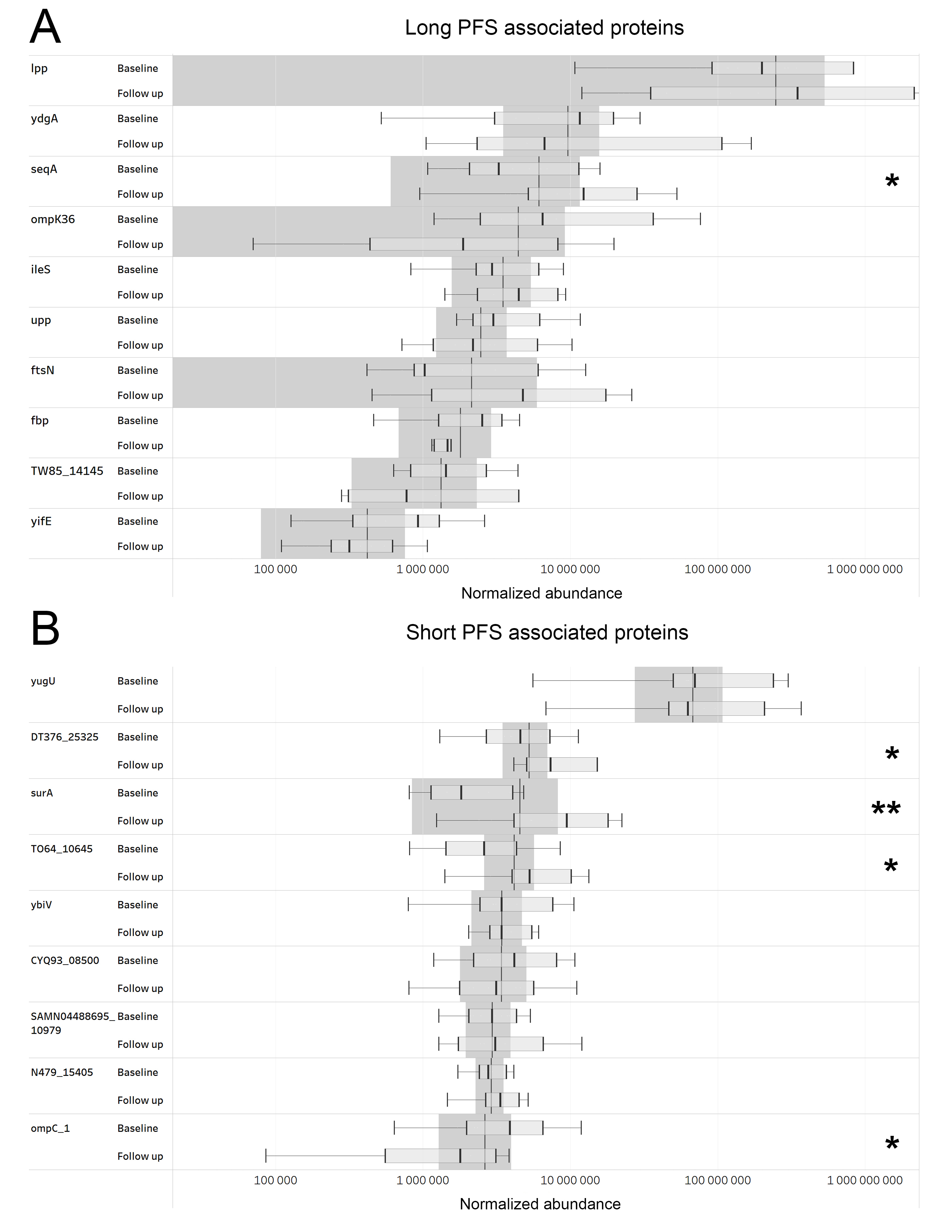


**SFig 6. Top bacterial urine proteins: baseline vs follow up comparison.** Bar charts show relative abundances of the top 10 (9) bacterial urine proteins associated with long (A) and short (B) PFS relative to the time of sampling (baseline vs follow up). Statistical comparison is performed with the Wilcoxon rank-sum (WRS) test. **p < 0.05, **p < 0.01, ***p < 0.001*


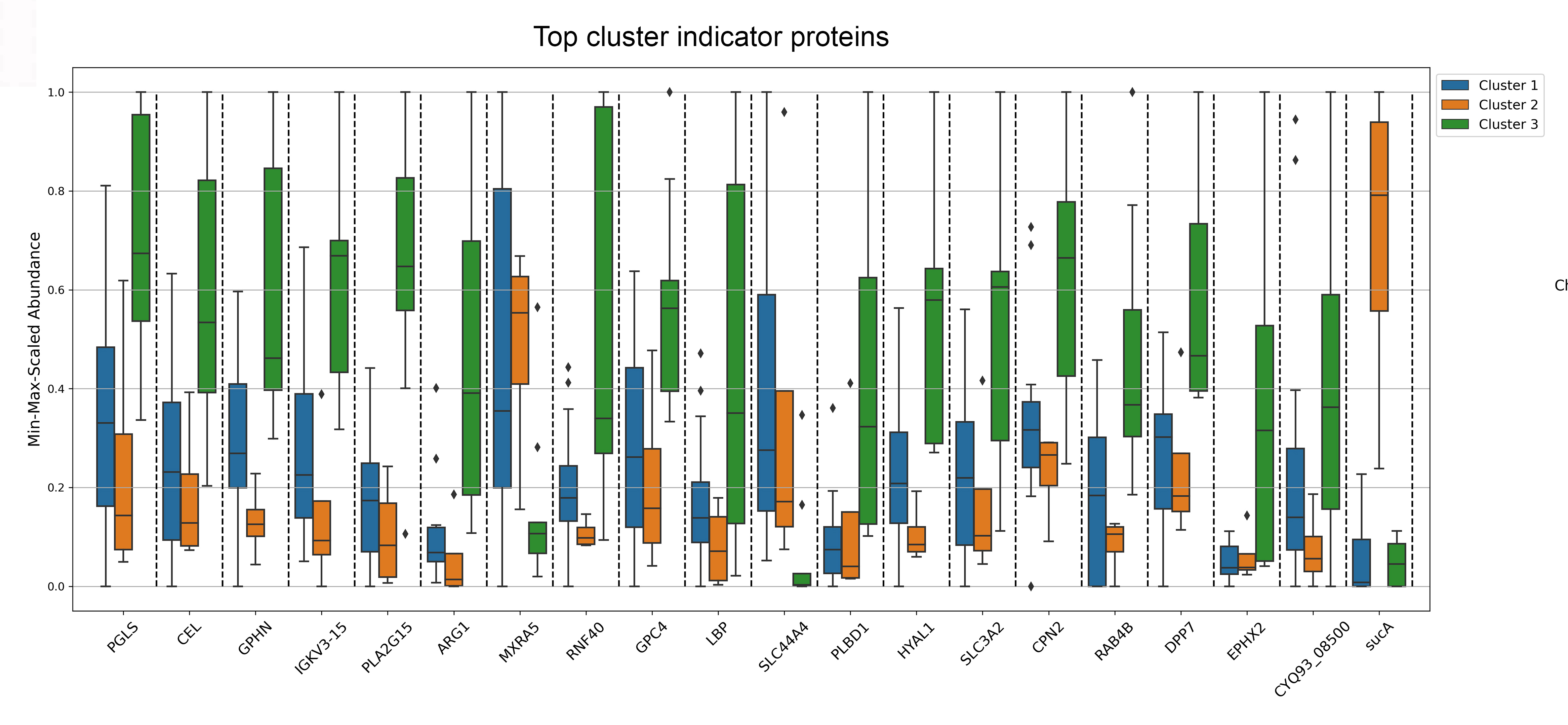


**SFig 7. Top 20 most discriminatory human/bacterial EV proteins determining clusters.**


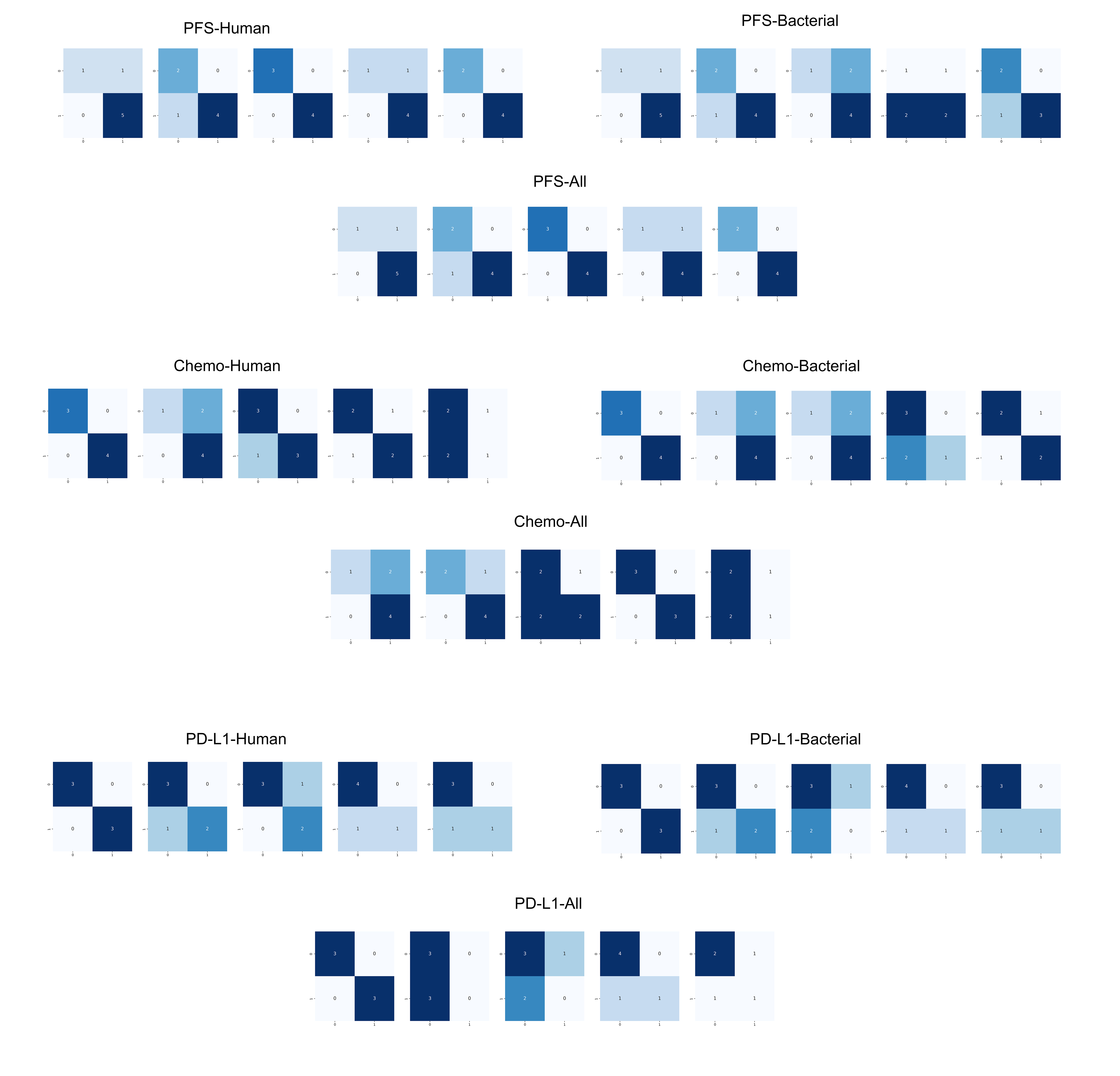


**SFig 8. Confusion matrices for RF machine learning algorithm**

**SUPPLEMENTARY TABLES**

**STable 1. Inclusion and Exlusion criteria of our study cohort**

| Inclusion criteria | Exclusion criteria |
| --- | --- |
| Eastern Cooperative Oncology Group (ECOG) Performance status 0 or 1 | Known driver mutation detected |
| Histologically or cytologically documented locally advanced or metastatic NSCLC not eligible for curative surgery and/or definitive thoracic radiotherapy or chemoradiotherapy, or metastatic Stage IIIB/IV non-squamous or squamous NSCLC | Symptomatic, severe central nervous system metastases |
| PD-L1 tumor expression > 1% | Positive test result for human immunodeficiency virus (HIV) |
| Measurable disease per Response Evaluation Criteria in Solid Tumors, Version 1.1 (RECIST v1.1) | Active hepatitis B or hepatitis C |
| Adequate hematologic and end-organ function | Suspected urinary tract infection within 2 weeks of sampling |
| Prior systemic treatment for metastatic NSCLC was allowed | Systemic antibiotic-treatment within 3 months of sampling |

**STable 2. Type of Immunotherapies administered to patients.** *P-values indicate Fischer’s exact tests. Distributions in different therapeutic groups are compared to the distribution of the whole cohort according to PFS (long vs short).*

| **Type of therapy** | **Long PFS**  N=22 (67%) | **Short PFS**  N=11 (33%) | **p-value** |
| --- | --- | --- | --- |
| Pembrolizumab monotherapy [n=10 (30%)] | n=7 (70%) | n=3 (30%) | >0.99 |
| Nivolumab monotherapy [n=16 (48%)] | n=10 (63%) | n=6 (37%) | >0.99 |
| Atezolizumab monotherapy [n=1 (3%)] | n=0 (0%) | n=1 (100%) | 0.352 |
| CHT+IO (pembrolizumab) combination^1^  [n=3 (9%)] | n=3 (100%) | n=0 (0%) | 0.537 |
| CHT+IO (durvalumab) combination^2^  [n=3 (9%)] | n=2 (67%) | n=1 (33%) | >0.99 |

^1^ IO with concurrent CHT: pembrolizumab + pemetrexed + carboplatin combination therapy,

^2^ IO with concurrent CHT: durvalumab + carboplatin+pemetrexed combination therapy
